# Deletion of AMPK minimizes graft-versus-host disease through an early impact on effector donor T cells

**DOI:** 10.1172/jci.insight.143811

**Published:** 2021-07-22

**Authors:** Darlene A. Monlish, Kevin J. Beezhold, Pailin Chiaranunt, Katelyn Paz, Nathan J. Moore, Andrea K. Dobbs, Rebecca A. Brown, John A. Ozolek, Bruce R. Blazar, Craig A. Byersdorfer

**Affiliations:** 1 Division of Blood and Marrow Transplantation and Cellular Therapies, Department of Pediatrics, University of Pittsburgh School of Medicine, Pittsburgh, Pennsylvania, USA.; 2 Division of Pediatric Blood and Marrow Transplantation, Department of Pediatrics, University of Minnesota, Minneapolis, Minnesota, USA.; 3 Department of Pathology, Anatomy and Laboratory Medicine, School of Medicine, West Virginia University, Morgantown, West Virginia, USA.

**Keywords:** Metabolism, Transplantation, Bone marrow transplantation, Fatty acid oxidation, Immunotherapy

## Abstract

Allogeneic hematopoietic stem cell transplantation is a viable treatment for multiple hematologic diseases, but its application is often limited by graft-versus-host disease (GVHD), where donor T cells attack host tissues in the skin, liver, and gastrointestinal tract. Here, we examined the role of the cellular energy sensor AMP kinase (AMPK) in alloreactive T cells during GVHD development. Early posttransplant, AMPK activity increased more than 15-fold in allogeneic T cells, and transplantation of T cells deficient in both AMPKα1 and AMPKα2 decreased GVHD severity in multiple disease models. Importantly, a lack of AMPK lessened GVHD without compromising antileukemia responses or impairing lymphopenia-driven immune reconstitution. Mechanistically, absence of AMPK decreased both CD4^+^ and CD8^+^ effector T cell numbers as early as day 3 posttransplant, while simultaneously increasing regulatory T cell (Treg) percentages. Improvements in GVHD resulted from cell-intrinsic perturbations in conventional effector T cells as depletion of donor Tregs had minimal impact on AMPK-related improvements. Together, these results highlight a specific role for AMPK in allogeneic effector T cells early posttransplant and suggest that AMPK inhibition may be an innovative approach to mitigate GVHD while preserving graft-versus-leukemia responses and maintaining robust immune reconstitution.

## Introduction

Allogeneic hematopoietic stem cell transplantation is a lifesaving treatment for hematologic disorders, but its application is limited by acute graft-versus-host disease (GVHD), where donor T cells attack and destroy tissues in the recipient liver, gut, skin, and lung (1). GVHD remains a prevalent clinical problem (2), and steroids, as the primary treatment for acute GVHD, encompass a myriad of side effects, including suppression of antiviral and antifungal immunity and increased relapse rates due to suboptimal graft-versus-tumor (GVT) responses (3–5). Furthermore, steroid-refractory GVHD portends a dismal prognosis, with mortality rates approaching 90% (6).

Novel therapies to impair GVHD-causing T cells are required, and metabolic modulation of T cells offers an innovative way to selectively target GVHD responses. Following transplantation, alloreactive T cells undergo dramatic reprogramming to increase their oxidative metabolism (7–9), enhance flux through the electron transport chain (ETC), and increase fatty acid oxidation (FAO) (10). Increased ETC flux is fundamental to alloreactive T cell survival, as inhibition of either complex I or complex V induces donor cell apoptosis and decreases GVHD severity (11, 12). These results are consistent with studies in solid organ transplantation, where metabolic inhibition decreases alloreactivity and prolongs graft survival (13). A clinical role for metabolic inhibition is also well established, with targeting of mammalian target of rapamycin (mTOR), which integrates cues for T cell activation, differentiation, and function (14), used for both GVHD prophylaxis and treatment (15).

AMP kinase (AMPK) is a highly conserved, heterotrimeric energy sensor with an α subunit supplying kinase activity and β/γ proteins providing stability and regulating substrate specificity. Multiple isoforms exist for each subunit, with AMPKα1 and α2 present in both mice and humans. AMPKα1 acts as the dominant catalytic subunit in naive mouse T cells (16), but whether AMPKα2 compensates in vivo in the absence of AMPKα1 is unknown (16, 17). In other tissues, AMPK becomes activated when intracellular ADP/ATP ratios rise, increasing oxidative metabolism and driving FAO (18, 19). In skeletal muscle, production of malonyl-CoA by acetyl CoA-carboxylase (ACC) allosterically inhibits carnitine palmitoyltransferase 1a (CPT1a), the rate-limiting enzyme of FAO (20). When AMPK becomes activated, it phosphorylates ACC, which reduces ACC activity, limits malonyl-CoA production, and frees CPT1a to increase FAO. AMPK also decreases mTOR signaling, through phosphorylation of tuberous sclerosis complex 2 (TSC2) and the mTORC1 binding partner Raptor (21, 22), and phosphorylates Unc51-like kinase 1 (ULK1) to facilitate early steps in autophagolysosome (APL) generation (23). Thus, perturbations in AMPK would be expected to decrease FAO, impair autophagy initiation, and increase mTOR signaling.

Despite growing knowledge of AMPK’s biology, defining an encompassing role for AMPK in T cells has proved elusive. Early studies demonstrated transient activation of AMPK following CD3 and Ca^2+^ stimulation (16), while systemic ablation of AMPKα1 increased lymphocyte susceptibility to mitochondrial inhibition (24). In later studies, global deletion of AMPKα1 increased T cell glycolysis and enhanced production of interferon-γ (IFN-γ) and interleukin 17a (IL-17a) (17). T cell–specific deletion of AMPKα1 did not affect primary T cell responses, but instead decreased CD8^+^ T cell numbers upon secondary challenge (25). More recently, AMPK was shown to be necessary in effector T cells (Teffs) during viral and bacterial challenges (26) and to drive oxidative metabolism in leukemic T cells (27). AMPK has also been implicated in regulatory T cell (Treg) development, given increased phosphorylation of AMPK under Treg culturing conditions in vitro and elevated percentages of Tregs following in vivo treatment with the indirect AMPK activator metformin (28, 29).

In many cases, a biological role for AMPK has been inferred from the relationship between metformin and AMPK activation (12, 28), but assigning causality can be difficult due to metformin’s effects independent of AMPK. In addition, T cells lacking both AMPKα1 and AMPKα2 (AMPK-dKO) remain uncharacterized, particularly in vivo. Here, using AMPK-dKO donor T cells, we demonstrate a selective necessity for AMPK in T cells during allogeneic, but not syngeneic, transplantation in a mechanism that defines an early role for AMPK in conventional Teffs.

## Results

### Alloreactive T cells increase AMPK activation during acute GVHD.

**Figure 1:**
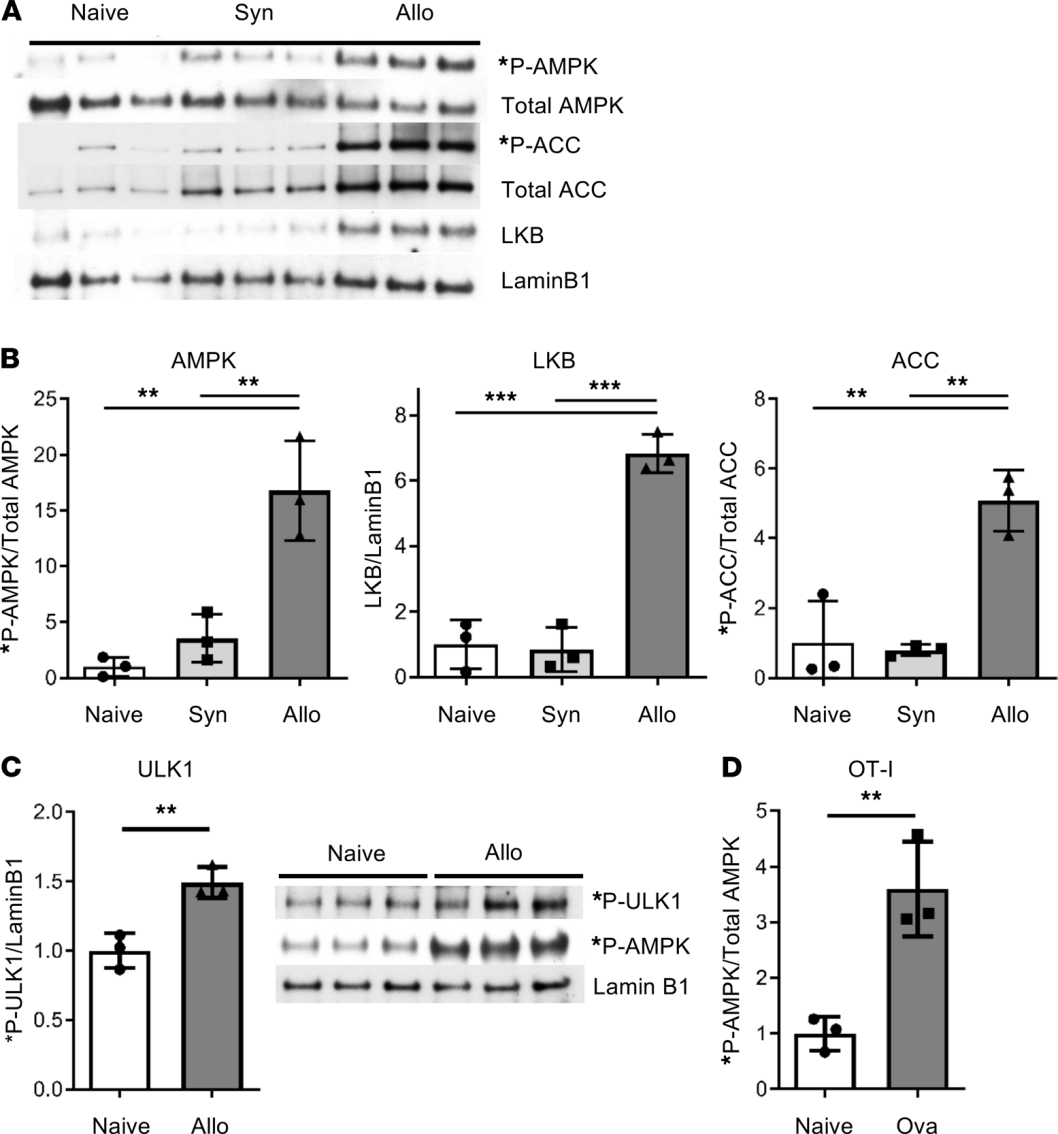
Alloreactive T cells selectively activate AMPK by day 7 posttransplant. (**A**) Two million CD45.1^+^ T cells and 5 × 10^6^ B6 bone marrow (BM) cells were transplanted into irradiated syngeneic (B6) or allogeneic (B6D2F1) recipients. On day 7 posttransplant, CD45.1^+^ donor T cells were flow-sorted and cell lysates immunoblotted for total and phosphorylated proteins in the AMPK pathway. Naive, donor T cells prior to transplantation. (**B**) Ratios of phosphorylated/total AMPK (left), liver kinase B1/Lamin B1 (middle), and phosphorylated/total ACC (right) were calculated based upon densitometry of blots in **A**. (**C**) In a separate experiment, day 7 donor cell lysates were blotted for phosphorylated ULK1 (*P-ULK1), with *P-ULK1/LaminB1 ratios calculated as above. In **A**–**C** *n* = 10 animals per condition pooled into 3 separate groups. (**D**) One million OT-I and 1 × 10^6^ OT-II cells were transplanted into irradiated CAG-OVA mice, followed by flow-sorting of OT-I cells on day 6 after BM transplantation. Immunoblotting compared phospho/total AMPK in pretransplant (naive) versus day +6 OT-I cells (*n* = 3). Data in each panel represent 3 or more independent experiments. **P < 0.01, ***P < 0.001.

Allogeneic T cells increase FAO by day 7 posttransplant (10), a finding that could be explained by increased AMPK activity, subsequent phosphorylation of ACC, and liberation of CPT1a. To test this possibility, CD45.1^+^ B6 T cells were transplanted into irradiated CD45.2^+^ B6xDBA2 F1 (B6D2F1) recipients in a major histocompatibility complex (MHC) mismatch model of GVHD. On day 7, donor T cells undergoing more than 8 divisions (i.e., CellTrace^lo^) were flow-sorted, and AMPK activity was evaluated by immunoblot. AMPKα activation (denoted by phosphorylation of Thr172) increased more than 15-fold in allogeneic CD4^+^ and CD8^+^ T cells but changed only minimally in syngeneic T cells (Figure 1, A and B). Furthermore, AMPK activation correlated with a 6.8-fold increase in levels of the upstream kinase liver kinase B1 (LKB) and was confirmed through enhanced phosphorylation of both ACC and ULK1 (Figure 1, B and C, and Supplemental Figure 1A). To determine if AMPK activity increased specifically in response to antigen, OT-I and OT-II T cells were cotransplanted into irradiated CAG-OVA mice (expressing OVA as a self-protein) and OT-I cells purified by flow-sorting on day 6 posttransplant. AMPK phosphorylation increased 3.5-fold in OT-I T cells on day 6 (Figure 1D and Supplemental Figure 1B; supplemental material available online with this article; https://doi.org/10.1172/jci.insight.143811DS1), demonstrating increased AMPK activation directly in antigen-responsive cells. Thus, allogeneic T cells induce LKB expression and increase AMPK activity in direct response to alloantigen.

### AMPK-dKO T cells maintain T cell development and in vitro proliferation.

**Figure 2:**
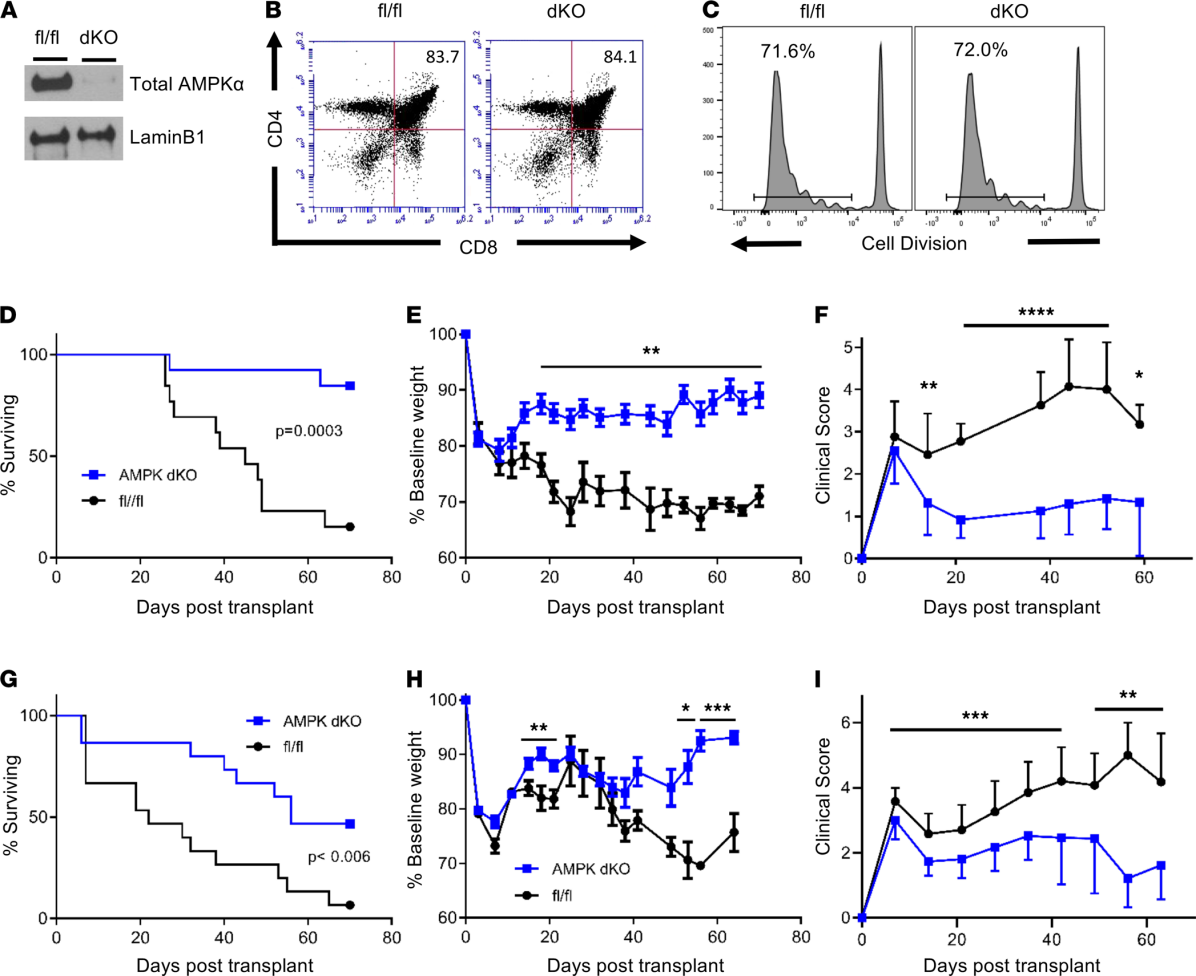
AMPK-dKO T cells develop normally but cause less GVHD. (**A**) TCRβ^+^ cells were flow-sorted from WT (fl/fl) or AMPK-dKO spleens and cell lysates blotted for total AMPKα. (**B**) Thymi were recovered from 8-week-old fl/fl or AMPK-dKO mice and stained for CD4 and CD8. (**C**) CD45.1^+^ fl/fl or AMPK-dKO T cells were labeled with CellTrace violet and placed in an MLR with B6D2F1 splenocytes, and division profiles were assessed at 72 hours. (**D**–**F**) To measure GVHD potential, 2 × 10^6^ fl/fl or AMPK-dKO T cells were transplanted into lethally irradiated C3.SW recipients and survival (**D**), weight loss (**E**), and clinical scores (**F**) measured to 10 weeks posttransplant (*n* = 16 mice/group in **D**–**F**). (**G**–**I**) Survival (**G**), weight loss (**H**), and clinical score (**I**) were similarly quantitated following allogeneic transplantation of fl/fl versus AMPK-dKO T cells into B6D2F1 recipients (major MHC mismatch model, *n* = 16 mice/group). Data represent 3 or more independent experiments. *P < 0.05, **P < 0.01, ***P < 0.001, ****P < 0.0001 by log-rank (Mantel-Cox) analysis for survival curves or by Student’s *t* test of individual weekly scores.

To test AMPK’s necessity in T cell responses, we intercrossed CD4Cre and AMPKα1^fl/fl^ AMPKα2^fl/fl^ strains of mice (30, 31) to create AMPK-dKO animals. Because Cre recombinase is expressed in double-positive thymocytes, this cross effectively deleted AMPKα1/α2 in all peripheral CD4^+^ and CD8^+^ T cells (Figure 2A) without affecting thymocyte percentages (Figure 2B), total thymocyte numbers, spleen cell numbers, or CD4^+^CD8^+^ ratios (Supplemental Figure 2, A–F). The one reproducible change was a minimal decrease in percentage of splenic TCRβ^+^ cells (Supplemental Figure 2D). Functionally, AMPK-dKO T cells proliferated equivalently to WT (fl/fl) T cells in an MLR (Figure 2C) and following stimulation by plate-bound CD3/CD28 antibodies (data not shown, dns). Thus, deletion of AMPKα1/α2 during thymic selection minimally impacted T cell development and produced donor cells capable of equivalent proliferation in vitro.

### Decreased severity of acute GVHD following transplantation of AMPK-dKO T cells.

To test the role of AMPK during GVHD, 2 × 10^6^ fl/fl or AMPK-dKO T cells were transplanted into irradiated C3H.SW (H2^b^) recipient mice in a minor antigen mismatch model of GVHD (32, 33). Mice receiving AMPK-dKO T cells experienced a distinct survival advantage (85% of recipients were alive at 10 weeks), with less weight loss and improved clinical scores (Figure 2, D–F). In contrast, recipients of fl/fl T cells experienced decreased survival (median of 45 days), and only 15% were alive on day 70. These findings were then replicated in the B6 into B6D2F1 model of GVHD, where the AMPK-dKO donor facilitated a similar survival advantage in the major MHC mismatch setting (Figure 2G), with concomitant improvements in clinical scores and lower overall weight loss, particularly at later time points (Figure 2, H and I). Thus, deletion of AMPK in donor T cells reproducibly minimized acute GVHD in 2 separate models.

### AMPK-dKO T cells retain robust antileukemic potential.

**Figure 3:**
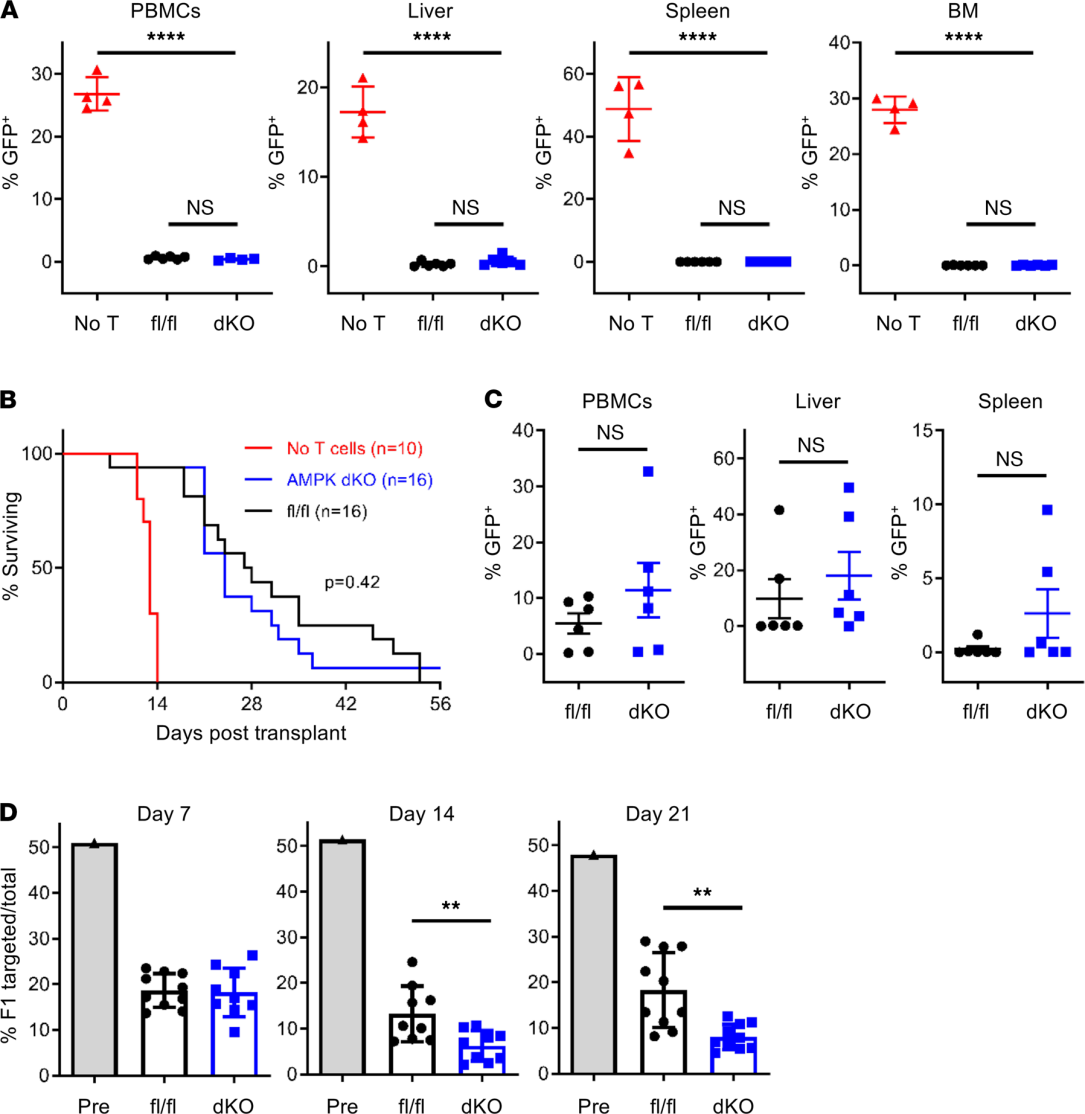
AMPK-dKO T cells preserve cytotoxicity and antileukemia responses. (**A**) One million fl/fl or AMPK-dKO T cells were transplanted with 10^5^ GFP^+^ p815 leukemia cells and 5 × 10^6^ TCD BM cells into irradiated B6D2F1 recipients. On day 13, the percentage of GFP^+^ leukemia cells was quantitated in the peripheral blood (PBMCs), liver, BM, and spleens of recipient mice. Mice receiving TCD BM and leukemia cells only (no T cells) served as controls for unrestricted leukemia growth (*n* = 4 recipients/group, with each experiment repeated twice). (**B**) Irradiated B6D2F1 mice were transplanted as in **A**, and survival was measured 10 weeks posttransplant (*n* = 10–16/group). (**C**) In a third cohort, the percentage of GFP^+^ cells was quantitated in the peripheral blood, liver, and spleens of recipient animals on day 28, the median point of survival (*n* = 6 recipients/group). (**D**) To measure in vivo cytotoxicity, B6D2F1 recipients were transplanted with fl/fl or AMPK-dKO T cells, then injected on day 6, 13, or 20 with a 1:1 mix of B6 (syngeneic) and B6D2F1 (allogeneic) splenocytes. One day later, the percentage of allogeneic B6D2F1 cells remaining was quantitated in the spleen (*n* = 8–10 mice/group). Data for all studies represent 2 or more independent experiments. **P < 0.01, ****P < 0.0001 by Student’s *t* test or for survival curves by log-rank (Mantel-Cox) analysis.

An ideal therapeutic intervention would mitigate GVHD while still preserving GVT responses. To measure AMPK’s necessity in antitumor immunity, 1 × 10^6^ fl/fl or AMPK-dKO donor T cells were transplanted with T cell–depleted (TCD) BM and a lethal dose of GFP^+^ p815 leukemia cells into irradiated B6D2F1 recipients. This reduced dose of allogeneic T cells rarely results in lethal GVHD (Supplemental Figure 3A), allowing for a more focused evaluation of AMPK effects on GVT responses and leukemia burden. We also intentionally used a robust number of leukemia cells to maximize potential discrimination between fl/fl and AMPK-dKO antitumor responses. Control mice, receiving leukemia cells and BM only, developed profound tumor burden by day 13 with significant infiltration into all organs examined. In contrast, both fl/fl and AMPK-dKO T cells orchestrated dramatic leukemia clearance from the peripheral blood, liver, spleen, and BM by day 13 (Figure 3A and Supplemental Figure 3B), indicating no difference in the early clearance of leukemia cells. We then followed recipient survival over time. Control mice receiving BM only lived a median of 13 days. T cells from AMPK-dKO donors extended median survival to 24 days, similar to fl/fl cells, with no difference in mortality between AMPK-dKO and fl/fl T cell groups (*P* = 0.57 by log-rank test, *n* = 16 mice/group) (Figure 3B). Death was likely tumor driven as all recipients experienced frank paralysis and/or had tumor nodules visible on necropsy. In addition, recovery of cells from the liver, spleen, and peripheral blood on day 28, near the point of median survival, indicated equivalent leukemia control between fl/fl and AMPK-dKO T cells (Figure 3C and Supplemental Figure 3C).

To evaluate GVT robustness in AMPK-dKO T cells, we repeated transplantations using a 4-fold reduction in donor T cells, 0.25 × 10^6^, but a similar number, 10^5^, of GFP^+^ p815 leukemia cells. Control mice, receiving leukemia and BM cells only, again developed profound tumor burden and survived a median of 13 days. Recipients of 1 × 10^6^ AMPK-dKO or fl/fl T cells survived a median of 27 and 28 days, respectively, with no significant difference in recipient mortality between groups (*P* = 0.79 by log-rank test, *n* = 6–7 mice/group). Importantly, 0.25 × 10^6^ T cells also conferred a survival advantage over control mice without T cells, but there was still no difference in median survival between AMPK-dKO and fl/fl groups at this lower dose of cells (*P* = 0.88 by log-rank test, *n* = 5–6 mice/group; Supplemental Figure 3D). Given that substantially fewer AMPK-dKO cells still improved survival equivalently to WT cells, we reduced donor T cell numbers even further (0.1 × 10^6^ cells/recipient) while maintaining 1 × 10^5^ p815 GFP^+^ leukemia cells. Following transplantation, mice were euthanized on day 13 and the percentage and number of GFP^+^ cells enumerated from the spleen, liver, and BM. Strikingly, leukemia clearance continued to be equivalent between recipients of AMPK-dKO and fl/fl cells with as few as 0.1 × 10^6^ donor T cells/recipient (*n* = 6–8 mice/group; Supplemental Figure 4, A–C). Together, these results demonstrate preservation of GVT effects in AMPK-dKO T cells over a wide range of donor T cell concentrations.

To evaluate in vivo cytotoxicity as another measure of GVT potential, transplanted B6D2F1 recipients were injected on day 6 with a 1:1 mix of B6/F1 splenocytes, and the percentage of F1 splenocytes was measured the following day. F1 percentages decreased from 50% pretransplant to 20% at 16 hours, regardless of donor T cell genotype (Figure 3D), indicating equivalent in vivo cytotoxicity between fl/fl and AMPK-dKO donor cells. Furthermore, the cytotoxic potential in recipients of AMPK-dKO cells was consistently better on days 14 and 21, demonstrating conclusively that absence of AMPK did not inhibit in vivo cytotoxic potential or impair effective antileukemia responses.

### AMPK-dKO T cells are recovered in lower numbers posttransplant.

**Figure 4:**
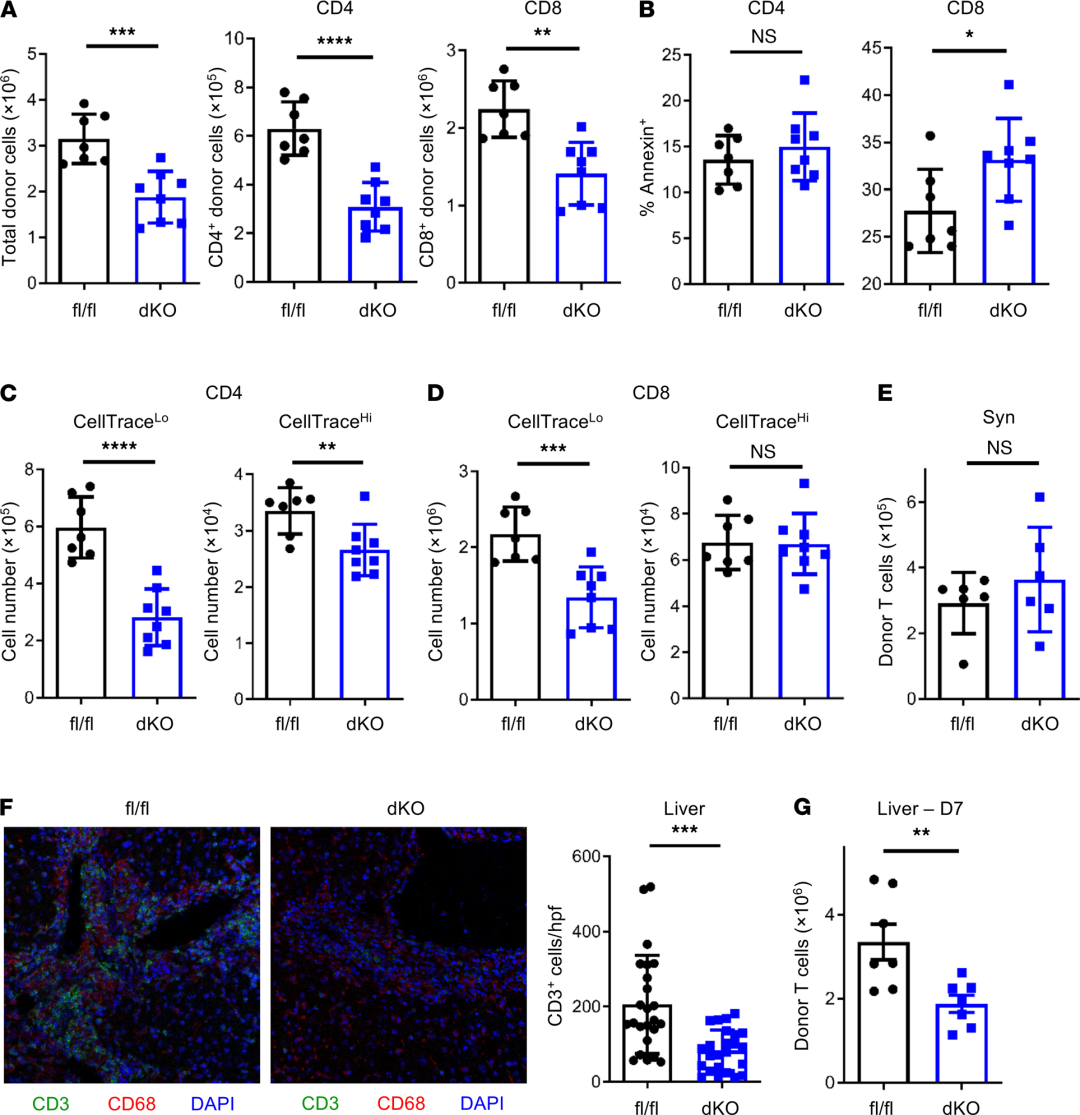
Fewer AMPK-dKO Teffs are recovered from allogeneic recipients. (**A**) Two million fl/fl or AMPK-dKO T cells were transplanted into B6D2F1 recipients, and total, CD4^+^, or CD8^+^ donor T cell numbers were quantitated in recipient spleens on day 7 posttransplant (*n* = 7–8 animals/group). Data represent more than 3 individual experiments. (**B**) Annexin V staining in CD4^+^ or CD8^+^ donor T cells recovered day 7 posttransplant (*n* = 7–8/group). (**C** and **D**) Division status was partitioned as CellTrace^lo^ (>8 divisions) or CellTrace^hi^ (≤8 divisions), and the number of donor T cells quantitated in each partition (*n* = 7–8/group). (**E**) Two million fl/fl or AMPK-dKO T cells were transplanted into lethally irradiated syngeneic B6 mice and donor T cells quantitated on day 7 (*n* = 6 recipients/group). Data are representative of 2 independent experiments. (**F**) B6D2F1 mice were transplanted as in **A**. On day 21 posttransplant, livers were flash-frozen and stained for CD3 (green), CD68 (red), and DAPI (blue) via immunofluorescence. Computer-assisted analysis quantitated the average number of CD3^+^ cells per high-powered field (hpf) from multiple confocal images (*n* = 8 mice/group with 3 images/mouse ≥24 images/group; 20× original magnification). (**G**) In a separate experiment, liver-associated donor T cells were enumerated on day 7 using Percoll separation followed by staining for TCRβ and CD45.1 (*n* = 7/group). Data in **G** are representative of 3 independent experiments. *P < 0.05, **P < 0.01, ***P < 0.001, and ****P < 0.0001 by Student’s *t* test.

CD4^+^ alloreactive T cells are required for initiation and propagation of acute GVHD, both in mouse models (34–36) and in clinical studies (37, 38). In the B6 into B6D2F1 model, disease is driven by CD4^+^ T cells but its severity is amplified by the presence of allogeneic CD8^+^ T cells. This model therefore allows tracking of AMPK’s effects in both CD4 and CD8 cells. To mechanistically understand the observed reductions in GVHD, donor T cells were enumerated on day 7 posttransplant. Forty percent fewer donor T cells were recovered following transplantation of AMPK-dKO cells, with reductions in both CD4^+^ and CD8^+^ populations (Figure 4A). A portion of the CD8^+^ reduction was attributable to enhanced apoptosis (Figure 4B), but AMPK necessity more closely correlated with cell division status. A majority of allogeneic T cells underwent more than 8 divisions to become CellTrace^lo^ by day 7 (Supplemental Figure 5). The remaining 8%–10% of cells undergoing less than 8 divisions reflected lymphopenia-induced proliferation (hereafter CellTrace^hi^). We then asked whether CellTrace^lo^ or CellTrace^hi^ cells were more affected by AMPK deficiency. There was a larger decrease in CellTrace^lo^ CD4^+^ T cells lacking AMPK compared with CellTrace^hi^ cells (Figure 4C, left versus right panel). In CD8^+^ T cells this distinction was absolute, where only divided (CellTrace^lo^) cells lacking AMPK decreased in number. From these data, we hypothesized that AMPK would be irrelevant during syngeneic transplantation, where the majority of proliferation is lymphopenia induced. To test this idea, 2 × 10^6^ fl/fl or AMPK-dKO T cells were transplanted into irradiated, syngeneic B6 animals and T cells enumerated on day 7. As expected, AMPK-dKO and fl/fl T cells were recovered in equal numbers following syngeneic transplantation (Figure 4E), consistent with a necessity for AMPK specifically in antigen-responding T cells (26).

### Decreased pathology and reduced immune cell infiltration in GVHD target organs.

To examine target organ pathology, day 22 paraffin sections of liver and small intestine were scored by a pathologist in a blinded fashion (*n* = 9–10 mice/group). Recipients of AMPK-dKO T cells had lower liver pathology scores (portal inflammation, bile duct injury, and central perivenulitis, each on a scale of 0 to 3) (39) and a trend toward decreased apoptosis in the small intestine (Supplemental Figure 6, A and B). Pathologic changes in the liver correlated with diminished hepatic infiltrates, as 2.5-fold fewer AMPK-dKO CD3^+^ cells were observed per high-powered field (Figure 4F) and CD3^+^ percentages were 50% of those seen with fl/fl cells (Supplemental Figure 6C). Percoll separation of liver-associated mononuclear cells on day 7 supported this finding, with 35% fewer T cells recovered from the livers of recipients of AMPK-dKO T cells at this early time point (Figure 4G). In total, AMPK-dKO T cells were recovered in lower numbers early posttransplant and induced less pathology in GVHD target organs.

### Classic metabolic pathways are unaffected by AMPK loss.

**Figure 5:**
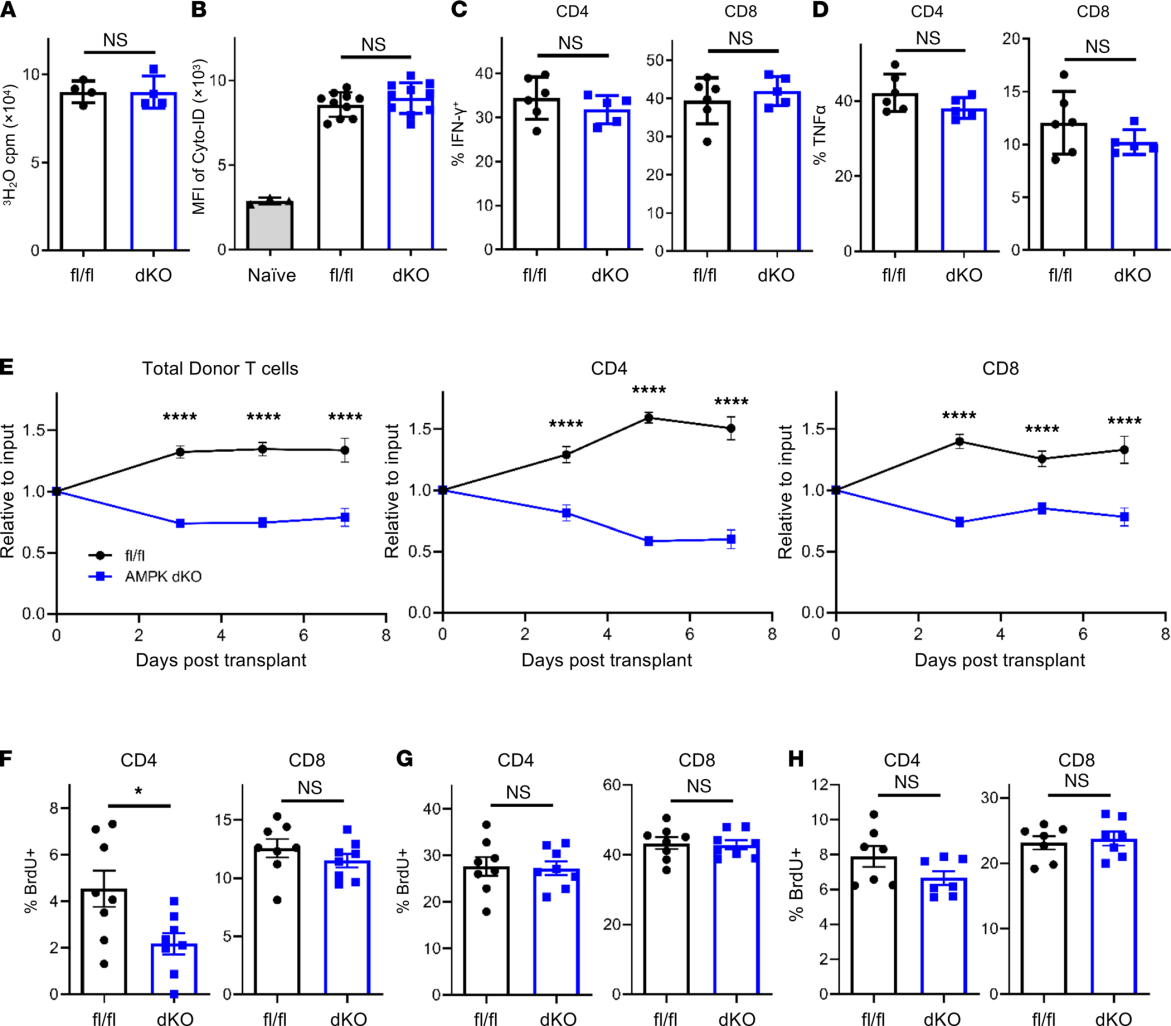
Early decreases in AMPK-dKO T cells are independent of changes in fat oxidation or autophagy. (**A**) WT (fl/fl) and AMPK-dKO T cells were enriched from allogeneic recipients on day 7 posttransplant and cultured overnight with ^3^H-palmitate, and ^3^H_2_O production was quantitated at 16 hours (*n* = 10–12 recipients pooled into 4 independent sets/group). (**B**) APL formation was quantitated with CYTO-ID staining prior to transplantation (naive), or in fl/fl versus AMPK-dKO donor T cells recovered on day 7 posttransplant (MFI, *n* = 3 mice/group for naive and 10 mice/group for fl/fl and AMPK-dKO). (**C** and **D**) Day 7 T cells were stimulated for 4 hours with PMA and ionomycin in the presence of brefeldin A, followed by flow cytometry determination of IFN-γ^+^ (**C**) and TNF-α^+^ (**D**) in CD4^+^ and CD8^+^ T cells (*n* = 5–6 mice/group). (**E**) One million congenically marked fl/fl donor T cells (CD90.1CD90.2) were combined with 1 × 10^6^ AMPK-dKO T cells (CD90.2) and cotransplanted into irradiated B6D2F1 recipients. On days 3, 5, and 7 posttransplant, the percentage of fl/fl versus AMPK-dKO T cells was compared with the original input percentages (*n* = 7–8 mice/group at each time point). (**F**–**H**) Recipients in **E** were injected with BrdU 30 minutes prior to euthanization, and the percentage of BrdU^+^ donor cells was determined by flow cytometry on on day 3 (**F**), day 5 (**G**), and day 7 (**H**) (*n* = 7–8 mice/group at each time point). In each case, data in **A**–**H** are representative of at least 2 independent experiments. *P < 0.05 and ****P < 0.0001 by Student’s *t* test.

Prior to oxidation, long-chain fatty acids (LCFAs) require coupling to carnitine, transport across the mitochondrial membrane, and decoupling back to acyl-CoA. This process can be quantitated ex vivo by measuring conversion of the radiolabeled LCFA ^3^H-palmitate to ^3^H_2_O. Because AMPK drives fat oxidation in multiple cells and tissues (40), we hypothesized that AMPK-dKO T cells would oxidize less fat than fl/fl T cells. Unexpectedly, AMPK-dKO T cells recovered on day 7 oxidized ^3^H-palmitate at rates equivalent to fl/fl cells (Figure 5A). Furthermore, this equivalence held despite varied ^3^H-palmitate exposure, additional TCR stimulation, and limitation of extracellular nutrients (dns).

AMPK could play other metabolic roles in alloreactive cells. During autophagy, AMPK phosphorylates ULK1 to facilitate APL formation (23). However, despite increased autophagy in allogeneic T cells (Supplemental Figure 7, A and B), neither APL formation, nor light chain 3 modification, decreased in cells lacking AMPK (Figure 5B and Supplemental Figure 7C). As AMPK can phosphorylate TSC2 and Raptor to decrease mTOR activity, deficiency of AMPK might be expected to increase mTOR signaling and heighten S6 phosphorylation (p-S6) (21, 22). However, p-S6 remained equivalent in day 7 cells, regardless of whether donor cells were AMPK-dKO or fl/fl (Supplemental Figure 7D). Finally, AMPK deficiency could skew cells from a pathogenic T helper type 1 (Th1) response toward a more tolerogenic Th2 phenotype. However, AMPK-dKO T cells expressed equivalently high levels of IFN-γ and TNF-α (Figure 5, C and D), with similar moderate to low levels of IL-4, IL-17, and IL-10 (Supplemental Figure 8, A–C). These latter data are consistent with preserved IFN-γ and IL-17 production observed in AMPKa1^–/–^ T cells during viral infections (26) and together suggest that improvements in GVHD following transplantation of AMPK-dKO T cells are not driven by changes in the canonical AMPK-related pathways of fat oxidation, autophagy, or mTOR signaling.

### AMPK deficiency decreases donor T cell numbers prior to day 3.

Lower numbers of AMPK-dKO T cells on day 7 could be explained by an early decrease in donor T cell proliferation. To test this idea in a cell-intrinsic manner, competitive transplants were performed by combining 1 × 10^6^ congenically marked fl/fl T cells (CD45.1^+^, CD90.1^+^CD90.2^+^) with 1 × 10^6^ AMPK-dKO T cells (CD45.1^+^, CD90.2^+^), followed by transplantation into irradiated B6D2F1 animals. Bromodeoxyuridine (BrdU) was administered 30 minutes prior to donor T cell recovery on days 3, 5, and 7 (experimental setup in Supplemental Figure 9, A and B).

Surprisingly, the percentage of AMPK-dKO T cells had already decreased by day 3 posttransplant, with CD8^+^ percentages remaining consistently low over all time points examined. AMPK-dKO CD4^+^ cells declined further between days 3 and 5 (Figure 5E). Peak proliferation occurred on day 5, but strikingly there was no difference in CD8^+^ T cell proliferation at any time point examined (Figure 5, F–H). In contrast, fewer AMPK-dKO CD4^+^ were proliferating on day 3, but AMPK-dKO CD4^+^ proliferated equivalently to fl/fl cells on days 5 and 7. Very few liver-associated cells were recovered on day 3, but AMPK-dKO percentages decreased steeply by day 5 and remained low (Supplemental Figure 10A), with no difference in proliferation of liver-associated AMPK-dKO T cells on either day 5 or 7 (Supplemental Figure 10, B and C). To press this finding further, fl/fl and AMPK-dKO T cells were transplanted into individual recipient mice with cells recovered on day 3. Again, AMPK-dKO T cells were recovered in lower percentages (Supplemental Figure 11A) but with equivalent donor T cell proliferation between AMPK-dKO and fl/fl cells (Supplemental Figure 11B). Together, these data indicate that both CD4^+^ and CD8^+^ AMPK-dKO T cells decrease in number in a cell-intrinsic manner prior to day 3, compounded by an early impairment in AMPK-dKO CD4^+^ T cell proliferation.

### CD4^+^FoxP3^+^ cells increase in recipients of AMPK-dKO cells.

**Figure 6:**
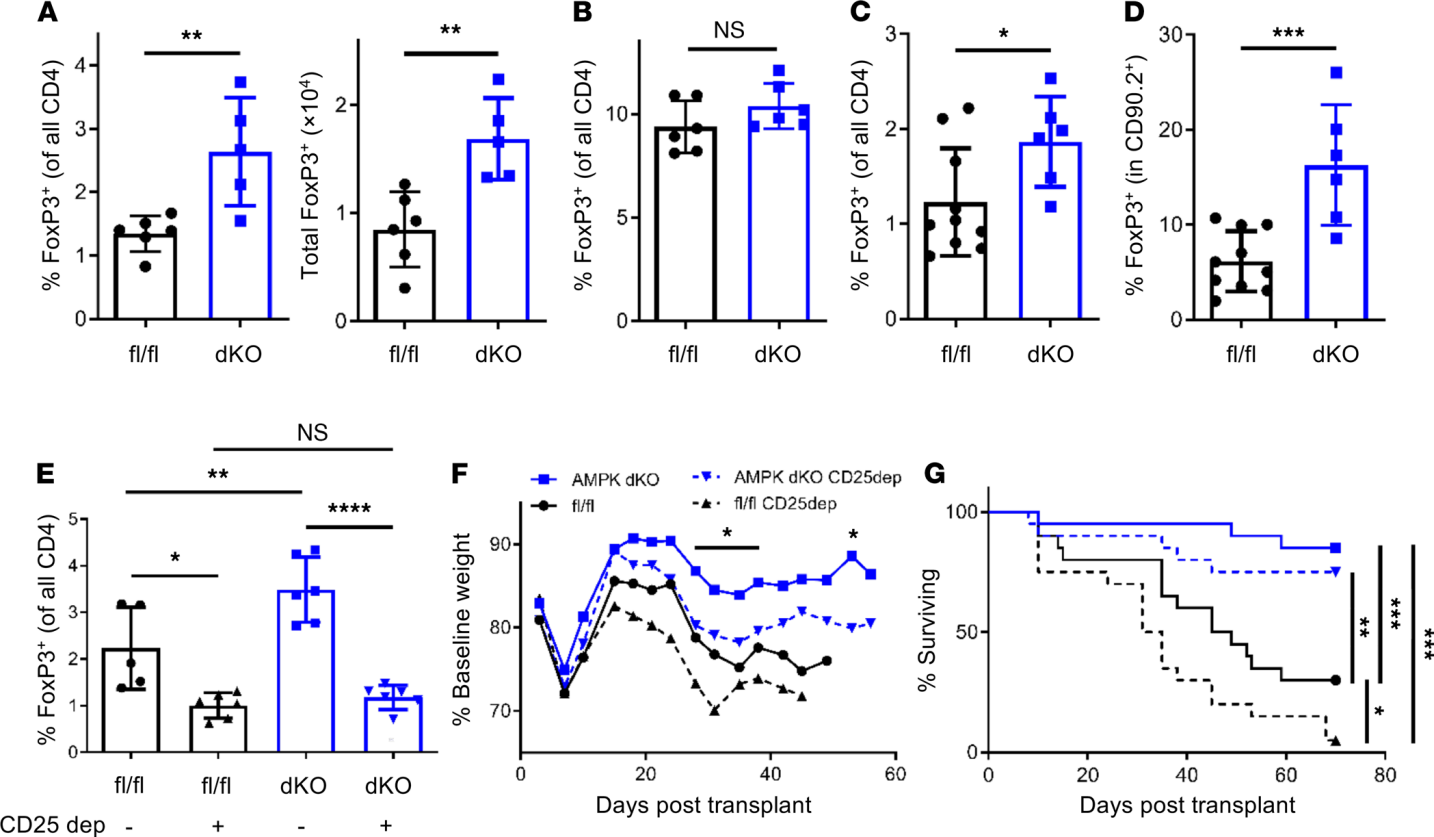
Donor Tregs are dispensable for AMPK-dKO–derived benefits. (**A**) WT or AMPK-dKO donor T cells were transplanted into B6D2F1 recipients, and the percentage (left) and total number (right) of splenic FoxP3^+^ cells were quantitated in allogeneic recipients on day 7 posttransplant (*n* = 8 mice/group). (**B**) Percentages of FoxP3^+^ cells in the donor inoculum from fl/fl versus AMPK-dKO donors (*n* = 4 mice/group). (**C** and **D**) Conventional fl/fl or AMPK-dKO T cells (CD90.1^+^CD90.2^+^) were mixed with CD90.2^+^ WT Tregs and cotransplanted into irradiated B6D2F1 mice. On day 7 posttransplant, FoxP3^+^ percentages were quantitated in all donor CD4^+^ T cells (**C**), or within the CD4^+^CD90.2^+^ subgroup (**D**). *n* = 6–10 mice/group. (**E**–**G**) WT or AMPK-dKO donor T cells, with or without CD25^+^ cell depletion, were transplanted into B6D2F1 recipients, and the percentage of FoxP3^+^ cells was measured on day 7 (in **E** *n* = 5–6 mice/group). Weight loss (**F**) and survival (**G**) were measured in a second cohort of recipients 10 weeks posttransplant (*n* = 20 mice/group combined from 2 independent experiments). Asterisks in **F** refer to statistically significant differences between AMPK-dKO donors with or without CD25 depletion. All experiments were replicated at least twice. *P < 0.05, **P < 0.01, ***P < 0.001, and ****P < 0.0001 by 1-way ANOVA followed by Tukey’s multiple comparisons test (**E**) or Student’s *t* test (all others). Survival curves were compared by log-rank (Mantel-Cox) analysis.

Previous reports have suggested that AMPK directly impacts Treg formation (28). Given that changes in GVHD are commonly attributed to differences in Treg number or function, underscored by the fact that exogenous Treg administration can prevent GVHD (41–44), we sought to understand the impact of AMPK deficiency on Tregs in the posttransplant environment. WT and AMPK-dKO T cells were transplanted into B6D2F1 hosts and Tregs enumerated on day 7. Surprisingly, both the percentage and number of Tregs increased 2-fold in recipients of AMPK-dKO T cells (Figure 6A). This was despite equivalent percentages and function of Tregs in the donor inoculum (Figure 6B and Supplemental Figure 12, A and B). Changes in Treg percentages could result from either cell-intrinsic changes or the influence of conventional cells (Tcons) on Treg stability. To differentiate between these possibilities, congenic fl/fl and AMPK-dKO Tcons (CD90.1^+^CD90.2^+^) were cotransplanted with WT (CD90.2^+^) Tregs and the percentage of FoxP3^+^ cells quantitated on day 7 (Supplemental Figure 13). WT Tregs were recovered in higher percentages if cotransplanted with AMPK-dKO Tcons (Figure 6C), and a greater percentage of original CD90.2 Tregs remained FoxP3^+^ on day 7 in the presence of AMPK-dKO Tcons (Figure 6D). These results demonstrate that the increased Treg percentage and number seen following transplantation of AMPK-dKO cells are independent of the AMPK status in Treg and instead depend on the genotype of the accompanying effector cell.

### Donor Tregs are dispensable for AMPK-dKO–derived benefits.

We next asked whether donor Tregs were integral to the benefits observed in the absence of AMPK. In the mouse, more than 90% of thymus-derived Tregs are CD25^+^ (45, 46), allowing for the comparison of donor grafts with or without pretransplant depletion of CD25^+^ cells. AMPK-dKO Treg-replete grafts increased FoxP3^+^ percentages on day 7, while pretransplant removal of CD25^+^ cells decreased day 7 Treg percentages to fl/fl levels (Figure 6E). We next asked whether Treg elimination equalized GVHD. WT and AMPK-dKO donor T cells, with or without CD25^+^ depletion, were transplanted into B6D2F1 recipients and monitored for 10 weeks. Transplantation of Treg-replete, AMPK-dKO grafts increased recipient survival (85% versus 30% alive at week 10, *P* < 0.0005), with decreased weight loss and lower clinical scores compared with recipients of Treg-replete fl/fl grafts (Figure 6, F and G, and Supplemental Figure 14A). CD25^+^ depletion in fl/fl donors exacerbated GVHD severity, with shorter median survival and a trend toward increased weight loss. Mice receiving CD25^+^-depleted, AMPK-dKO grafts fared much better, outperforming even Treg-replete fl/fl donors. Thus, pretransplant removal of donor Tregs does not obviate the benefits seen with transplantation of AMPK-dKO cells.

These Treg findings were then verified in a second model using AMPK-dKO × FoxP3^DTR^ mice, where FoxP3-driven expression of the diphtheria toxin receptor (DTR) sensitizes Tregs to DT administration (47). Transplantation of allogeneic FoxP3^DTR^ cells followed by DT administration on days 0 and 1 decreased donor Tregs more than 10-fold in recipient spleen and lymph nodes (Supplemental Figure 14, B and C). However, despite significant Treg elimination, transplantation of AMPK-dKO donor cells still improved survival and decreased weight loss in recipient animals compared with fl/fl cells (Supplemental Figure 14, D and E). Thus, donor Tregs are dispensable for improvements following transplantation of AMPK-dKO cells, which instead are attributed to early differences in non-Treg effector cells.

### Human allogeneic T cells activate AMPK.

**Figure 7:**
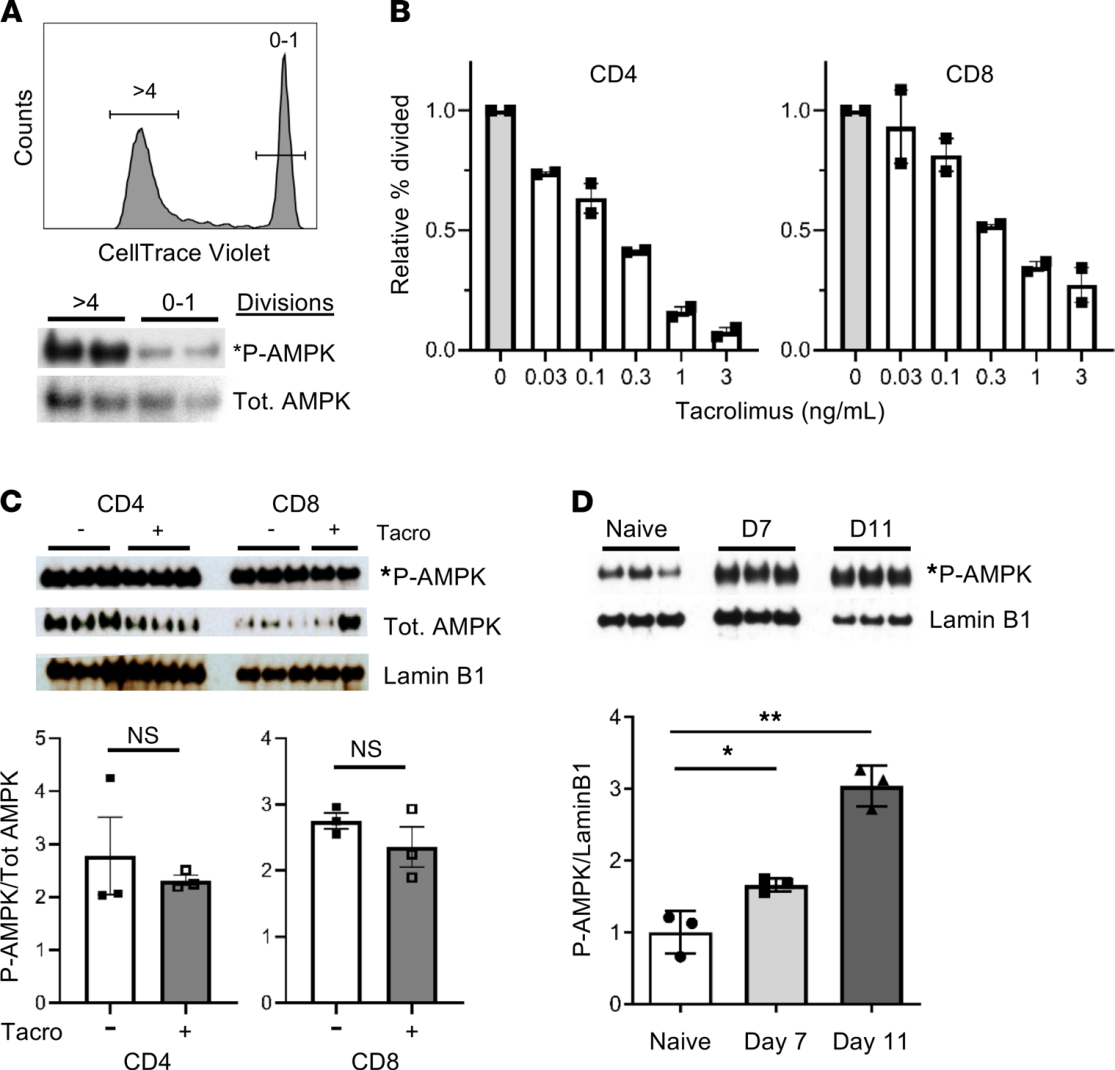
AMPK is activated in human allogeneic T cells. (**A**) T cells from 2 healthy donors were labeled with CellTrace and placed in an MLR with Mitomycin-C–treated allogeneic stimulator cells. At 96 hours, donor T cells undergoing either 0–1 or more than 4 cell divisions were flow-sorted and cell lysates immunoblotted for total and p-AMPK (*n* = 2/group). (**B**) Peripheral blood T cells were plated with Mitomycin-C–treated allogeneic stimulator cells for 96 hours with varying concentrations of tacrolimus, followed by comparing divided donor T cell percentages in the treated versus untreated groups (*n* = 2). Data are representative of results from 4 independent donor/stimulator pairs in 2 separate experiments. (**C**) T cells were harvested, labeled with CellTrace, and placed into an allogeneic MLR ± 0.3 ng/mL tacrolimus. After 6 days, cells undergoing more than 2 divisions were flow-sorted, and AMPK activation was assessed by immunoblot analysis for p-AMPK (*n* = 2–3 responder/stimulator pairs). Bar graphs represent densitometry measurements from a second technical replicate where n = 3 responder/stimulator pairs. (**D**) Ten million human PBMCs were transplanted into lightly irradiated NSG mice, and human T cells were recovered on day 7 and 11 posttransplant, followed by immunoblotting for AMPK activity in CellTrace^lo^ cells (*n* = 3 mice for naive or 6–8 recipients/time point pooled into 3 individual sets, days 7 and 11). *P < 0.05, **P < 0.01 by Student’s *t* test.

To establish whether similar AMPK activation occurs in human cells, T cells were enriched from healthy donors, labeled with CellTrace, and placed into an allogeneic MLR. At 96 hours, cells were flow-sorted based on CellTrace status and protein lysates analyzed for phosphorylation of AMPK. Ratios of p-/total AMPK increased more than 50% in human T cells undergoing more than 4 divisions compared with cells with minimal proliferation (Figure 7A). We then sought to assess the translational implications of this result. Calcineurin inhibitors (CNIs), such as tacrolimus, are used clinically as GVHD prophylaxis. To assess the effect of CNIs on AMPK activation, we conducted MLRs in the presence or absence of tacrolimus. We first determined that tacrolimus at 0.3 ng/mL inhibited proliferation of both CD4^+^ and CD8^+^ human T cells by 50% (Figure 7B). Human T cells were then harvested from healthy donors, labeled with CellTrace, and placed with 3 individual pairs of allogeneic non-T cells with/without tacrolimus at 0.3 ng/mL. On day 6, dividing CD4^+^ and CD8^+^ T cells were flow-sorted based on CellTrace status (>2 divisions) and protein lysates assessed for AMPK phosphorylation by immunoblot. Equivalent AMPK activation occurred in divided CD4^+^ and CD8^+^ T cells regardless of tacrolimus exposure, with equal p-/total AMPK ratios with/without tacrolimus (Figure 7C). These MLR data demonstrate that while prophylactic CNI administration may lower the percentage of responding cells, T cells that still undergo alloantigen-driven proliferation in the presence of a CNI continue to increase AMPK activation to a similar degree.

To confirm AMPK activation in human T cells in vivo, 10^7^ human peripheral blood mononuclear cells (PBMCs) were labeled with CellTrace and injected into lightly irradiated NOD-scid-IL2R-γ^null^ mice in a xenogeneic model of GVHD (48). AMPK activation increased in dividing T cells by day 7, with a further increase by day 11, at which time p-/total AMPK ratios were 3-fold higher than in naive T cells (Figure 7D). Thus, AMPK activation increases in human T cells both in vitro and in vivo, similar to what is found in murine allogeneic T cells.

## Discussion

AMPK drives metabolic reprogramming in a variety of cells (18, 19) and plays a pivotal role in T cell responses in vivo and in vitro (17, 24–26). However, the full scope of AMPK’s influence on immune reactions is still evolving. In this study, deletion of AMPKα1/α2 in donor T cells decreased allogeneic donor T cell numbers in a cell-intrinsic manner prior to day 3 posttransplant, in the process decreasing GVHD while simultaneously sparing lymphopenia-driven immune reconstitution and GVT effects. These results are consistent with reports showing reduced recovery of AMPKα1^–/–^ CD8^+^ T cells following in vivo inflammation but equivalent retrieval upon transfer into immunodeficient hosts (26). Together these reports argue that AMPK-dKO T cells decrease in number in response to antigen but not in response to growth signals from a lymphopenic environment. AMPK interacts with multiple signaling pathways, which could explain the decreased GVHD potential of AMPK-dKO cells (49–51). However, significant changes occur in this model within 3 days of transplantation, suggesting an acute change in T cell behavior. In this light, it is known that AMPK becomes activated within 30 minutes of TCR stimulation (16), which might explain the rapid loss of donor T cells upon exposure to large quantities of antigen in vivo.

Multiple approaches have attempted to separate GVHD and GVT responses (reviewed in ref. 1), including limiting Th1 responses (52), blocking inflammatory cytokines (e.g., TNF-α) (53), and differentially impacting CD4^+^ or CD8^+^ responses (54–56). In both murine and clinical studies, CD4^+^ T cells are instrumental in driving GVHD while CD8^+^ T cells play a larger role in mediating GVT effects. In the current studies, AMPK deficiency had a greater impact on CD4^+^ T cells, with a larger decrease in CD4^+^ T cell numbers by day 7 (Figure 4, C and D) and impaired proliferation only in CD4^+^ T cells at early times posttransplant (Figure 5E). Because these changes were less prevalent in CD8^+^ T cells, this dichotomy could preferentially impact CD4-driven GVHD severity while preserving GVT responses. Secondly, while there was a substantial reduction in total AMPK-dKO T cells recovered from allogeneic recipients on day 7 (3 × 10^6^ versus 2 × 10^6^), 2 × 10^6^ donor T cells still represent a 5-fold increase in donor cell numbers compared with syngeneic transplantation. Thus, there likely remain adequate numbers of allogeneic cells to mediate GVT effects, but with enough of a reduction to prevent rampant GVHD. Reassuringly, GVT responses using AMPK-KO T cells were equivalent to WT T cells over a wide (10-fold) range of donor T cell doses, suggesting that even at very low numbers, T cells lacking AMPK exert leukemia control that is equivalent to WT cells. Third, the increased Treg/Teff ratios observed with AMPK-dKO transplantation are more likely to impair GVHD than GVT responses given that exogenous Tregs control alloreactive T cell expansion without compromising GVT activity (42). Finally, although AMPK-dKO T cells decreased in number, cytokines important for mediating GVT immunity (including TNF-α and IFN-γ) were produced by AMPK-dKO T cells at levels equivalent to WT cells, and recipients of AMPK-dKO T cells demonstrated improved cytotoxic capacity. This increase in cytotoxicity could certainly be due to decreased exhaustion of AMPK KO T cells. However, it is also known that GVHD promotes posttransplant immune dysfunction, through both production of inflammatory cytokines and attack on the thymus as well as the BM compartment (57–61). In support of these possibilities, we are actively investigating levels of antiinflammatory cytokines to determine if they have a role in promoting the increased cytotoxicity seen with transplantation of AMPK-deficient T cells. Regardless of the cause, equivalent cell proliferation and sustained cytotoxicity, following an early loss of the most pathogenic effector cells, represents a beneficial aspect of AMPK-targeted interventions and is reminiscent of other therapies where effector cells are preferentially targeted early in the disease course (62). In addition, as AMPK activation is antigen driven, it is likely that the cells most reactive to alloantigen will also be the most susceptible to AMPK-related inhibition.

Several publications have suggested that AMPK drives FAO in T cells (28, 63, 64). Thus, it was surprising that AMPK-dKO cells oxidized fat at rates equivalent to WT cells. This fact separates T cells from other oxidative cell types (e.g., hepatocytes), where FAO is driven by AMPK (40). Furthermore, these data suggest that other mechanisms exist to increase fat oxidation in alloreactive T cells, with drivers of CPT1a expression among the list of candidates (10, 65, 66). AMPK has also been implicated in Treg homeostasis and proliferation (28). However, Tregs existed in equal numbers in AMPK-dKO donors, with equivalent baseline suppressive function, and were recovered at increased frequencies from allogeneic recipients. This discordance with previous studies may relate to underlying metabolic differences between in vitro– and in vivo–derived Tregs (7) coupled with the challenge of using indirect agonists, such as metformin, to assign definitive roles to AMPK. Given that Treg progenitors develop prior to the double-negative stage of thymic selection, we cannot exclude a role for AMPK on Treg formation at earlier times in development, but it is clear from the current work that in vivo Treg formation does not require AMPK from the double-positive stage of thymic selection onward. Finally, the equivalent FAO and Treg production seen in AMPK-dKO cells in these studies reinforces the importance of verifying metabolic pathways in a context-dependent manner (67), a point underscored by our prediction that AMPK would also be important for both T cell autophagy and mTOR activity (68), neither of which is true under the conditions examined.

Allogeneic and xenogeneic human T cells activate AMPK both in vitro and in vivo, suggesting that AMPK inhibition may represent a novel approach to prevent or treat GVHD clinically. Furthermore, equivalent AMPK activation in the presence of tacrolimus demonstrates that AMPK inhibition will still have an important role in preventing GVHD, even in patients on CNI-based immunosuppression. Donor T cells could be genetically modified to decrease expression of AMPKα (e.g., through deletion mediated by CRISPR technologies) followed by transplantation into allogeneic recipients. This approach would lessen the GVHD potential of the transplanted cells while allowing lymphopenia-driven immune reconstitution and GVT immunity. Perhaps more intriguing, given the dramatic changes seen with AMPK-dKO cells by day 3 posttransplant, would be treatment of T cells ex vivo with a pharmacologic inhibitor of AMPK just prior to injection. Such treatment could transiently impair allogeneic T cell responses during this critical early window of GVHD pathogenesis but potentially avoid long-term consequences of AMPK deficiency, including impaired generation of memory T cells (25). There is also the possibility of systemically inhibiting AMPK in recipients posttransplant, an approach with the obvious advantage of a more standard clinical application. However, given the pleiotropic nature of AMPK and the many pathways it orchestrates, pharmacologic inhibition at a systems level could lead to many on-target, off-tissue effects. Thus, any possibility of systemic therapy would need to first define an AMPK inhibitor of high specificity followed by testing in animal models to determine if a therapeutic window exists in which GVHD is inhibited but AMPK-related side effects can be minimized.

In summary, absence of AMPK in donor T cells reduced GVHD while preserving T cell–mediated cytotoxicity and maintaining lymphopenia-driven immune reconstitution. These benefits were related to an early decrease in conventional Teff cells without an impact on canonical AMPK-related pathways of FAO, autophagy, or mTOR inhibition. These findings have broad implications for our understanding in vivo T cell metabolism, including a strong reconsideration for the role of AMPK in driving FAO in T cells and the impact of AMPK signaling on Treg generation/homeostasis. These findings also suggest that T cell inhibition of AMPK may serve as a clinically relevant way to prevent GVHD while maintaining robust physiologic immunity.

## Methods

### Mice.

C57BL/6 (B6, H2^b^) and B6 × DBA2 F1 (B6D2F1) mice were purchased from Charles River. C3.Sw (H2^b^), CD45.1 (B6.SJL-Ptprca^Pepcb/Boy^J), CD4cre [B6.Cg-Tg(Cd4-cre)^1Cwi/Bflu^J], CAG-OVA [C57BL/6-Tg(CAG-OVAL)^916Jen^/J], Foxp3^DTR^ [B6.129(Cg)-Foxp3^tm3(DTR/GFP)Ayr^/J], OT-I [C57BL/6-Tg(TcraTcrb)^1100Mjb^/J], OT-II [C57BL/6-Tg(TcraTcrb)^425Cbn^/J], NSG (NOD.*Cg-Prkdc^scid^Il2rgt^m1Wjl^*/SzJ), and Thy1.1 (B6.PL-Thy1^a^/CyJ) mice were purchased from The Jackson Laboratory. AMPKα1^fl/fl^ α2^fl/fl^ mice were a gift from Sean Morrison (31) (University of Texas Southwestern, Dallas, Texas, USA) and backcrossed to C57BL/6 mice for more than 6 generations. Both male and female mice were used interchangeably. Recipient animals were 8–12 weeks old, and donor animals 8–16 weeks old, at the time of transplantation. All animals were housed in a specific pathogen–free facility.

### BM transplantation.

Unless otherwise stated, B6D2F1 mice were conditioned with 1250 cGy total body irradiation in a split dose from an x-ray source (X-rad 320, Precision X-Ray Irradiation), followed by intravenous infusion of 5 × 10^6^ TCD B6 BM cells and 2 × 10^6^ CD45.1^+^ B6 T cells enriched via CD90.2^+^ selection (Miltenyi Biotec). For minor histocompatibility mismatch transplants, C3.SW mice were conditioned with 1100 cGy in a single dose. B6 mice were irradiated with 1100 cGy (split dose) for syngeneic transplants. For transplantation into OVA-bearing recipients, CAG-OVA mice were irradiated with 1000 cGy in a single dose, followed by administration of 5 × 10^6^ TCD B6 BM cells and 1 × 10^6^ each of Vα2/Vβ5^+^ OT-I and OT-II T cells. In xenogeneic GVHD experiments, NSG mice were irradiated with 250 cGy followed by administration of 1 × 10^7^ fresh human PBMCs. Transplanted mice were housed in sterile microisolator cages, given hyperchlorinated drinking water for 3 weeks after BM transplantation (pH 3.0), and humanely euthanized for moribund behavior or weight loss more than 30% from baseline. Posttransplant recipient mice were weighed twice weekly and clinical scores assessed weekly as previously described (69).

### Cell sorting and immunoblot.

For protein precipitations, 10^5^ TCR-β^+^CD45-1^+^ CD4^+^ or CD8^+^ cells were flow-sorted (FACSAria, BD Biosciences) directly into 10% trichloroacetic acid followed by centrifugation at 16,000*g* for 10 minutes at 4°C, 2 washes in acetone, and resuspension in solubilization buffer (9 M urea, 2% Triton X-100, 1% dithiothreitol) (70). Lithium dodecyl sulfate loading buffer (Invitrogen, Thermo Fisher Scientific) was added to solubilized protein samples, followed by heating to 70°C for 10 minutes, separation on Bis-Tris polyacrylamide gels (Invitrogen, Thermo Fisher Scientific), and transfer to PVDF membranes (MilliporeSigma). Immunoblotting was performed according to Cell Signaling Technologies protocols. Blots were stripped (1% sodium dodecyl sulfate, 25 mM glycine, pH 2) prior to reprobing. Antibodies used for immunoblotting are listed in Supplemental Table 1. Blots were developed with Super Signal West Femto chemiluminescence reagents (Thermo Fisher Scientific), detected on HyBlot CL film (Denville Scientific), scanned in grayscale, and cropped in Microsoft PowerPoint. For quantitation, scanned images were resized to contain the bands of interest, copied into ImageJ software (NIH, version 1.44o), inverted, and densitometry quantitated in an area encompassing the largest band, followed by quantitation of all subsequent bands using the same 2-dimensional area.

### Flow cytometry.

Flow cytometry was performed as previously described (10). In brief, spleens and lymph nodes were pressed through a 70 μm strainer to generate a single-cell suspension, preincubated with 1G12 antibody to block nonspecific Fc binding, stained with directly conjugated antibodies for 15 minutes at 4°C, and washed twice. Antibodies and other reagents used for flow cytometry are listed in Supplemental Tables 2 and 3. Staining and washes were performed in PBS with 2% fetal bovine serum (FBS). FoxP3 staining was performed with an intracellular staining kit and according to manufacturer instructions (eBioscience, Thermo Fisher Scientific). Apoptosis was measured via annexin V–488 staining (Invitrogen, Thermo Fisher Scientific) for 15 minutes at room temperature in annexin staining buffer (BD Biosciences). APL formation was assessed using a CYTO-ID detection kit (Enzo Life Sciences). In some cases, transplanted animals were injected with BrdU 30 minutes prior to euthanization and samples processed according to manufacturer’s instructions (BioLegend 370706). Flow data were captured on a Fortessa analyzer (BD Biosciences) and evaluated using FlowJo software (version 10.1, Tree Star).

### Fatty acid oxidation.

Ex vivo FAO was performed as previously described (10). Briefly, 10^6^ day 7 T cells were flow-sorted from allogeneic recipient mice and plated for 16 hours in low-glucose DMEM (1000 mg/L glucose, Invitrogen, Thermo Fisher Scientific) with 10% FBS containing 15 mCi ^3^H-palmitate (PerkinElmer) conjugated to fatty acid–free BSA (MilliporeSigma) with/without 100 μM etomoxir. ^3^H_2_O production was quantitated by running supernatants through anion exchange columns (Evergreen Scientific) loaded with Dowex1x8 (MilliporeSigma). We calculated β-oxidation as the difference in counts per minute with or without etomoxir (71).

### In vivo cytotoxicity and DT treatment.

B6D2F1 mice were transplanted with fl/fl or AMPK-dKO T cells and on day 6, 13, or 20 posttransplant administered a 1-to-1 mix of B6 and B6D2F1 splenocytes (labeled with 5 versus 0.5 μM CellTrace violet from Invitrogen, Thermo Fisher Scientific, respectively). The following day, percentages of remaining B6D2F1 splenocytes (of all CellTrace-positive cells) were quantitated in the spleens of recipient animals. For Treg depletion studies, recipients of WT or AMPK-dKO Foxp3^DTR^ cells received 50 μg/kg DT (MilliporeSigma) via intraperitoneal injection on days 0 and +1 posttransplant.

### Mixed leukocyte reactions and tacrolimus treatment.

T cells were purified from human PBMCs via column-based selection, labeled with CellTrace violet, and placed at 3 × 10^5^/well in culture with 3 × 10^5^ allogeneic PBMCs or non-T cell antigen-presenting cells pretreated with 25 μg/mL Mitomycin-C. MLRs were performed on 96-well flat or round-bottom plates in AIM V media (Thermo Fisher Scientific) supplemented with 5% Immune Cell SR (Thermo Fisher Scientific). Cultures were assessed at 96 hours for cell division status and subsequently flow-sorted. For MLRs in the presence/absence of tacrolimus (MilliporeSigma), cultures were exposed to 0.3 ng/mL and assessed for cell division status after 6 days.

### Statistics.

Graphing and statistical analysis were performed using GraphPad Prism for Windows (version 9.0.1). Unpaired 2-tailed Student’s *t* test, log-rank (Mantel-Cox) test, or 1-way ANOVA was used to determine statistical significance. Unless noted otherwise, data are displayed as mean ± SEM. In all cases, *P < 0.05, **P < 0.01, ***P < 0.001, and ****P < 0.0001.

Additional method details are in Supplemental Methods.

### Study approval.

All animal studies were approved and carried out according to Institutional Animal Care and Use Committee guidelines from the University of Pittsburgh. All studies on human cells were designated exempt status by the University of Pittsburgh Institutional Review Board.

## Author contributions

DAM, KJB, and PC designed and performed experiments, analyzed data, and reviewed the manuscript. KP designed and performed experiments and analyzed data. NJM, AKD, and RAB performed experiments and analyzed data. JAO reviewed pathology slides, assigned scores in a blinded fashion, and reviewed the manuscript. CAB drafted the manuscript with assistance from DAM. BRB and CAB designed the studies, interpreted and analyzed data, and critically revised the final manuscript. Authorship order among coauthors was assigned based on percentage contribution to the final manuscript, intellectual involvement, and role in responding to reviewers’ inquiries.

## Supplementary Material

Supplemental data

## Version 1 07/22/2021

Electronic publication
