# Supplementary material for: Deletion of AMPK minimizes graft-versus-host disease through an early impact on effector donor T cells
Source: JCI Insight. 2021 Jul 22;6(14):e143811. doi: 10.1172/jci.insight.143811 (PMC8410053; doi:10.1172/jci.insight.143811)
Supplement: Supplemental data [file jciinsight-6-143811-s034.pdf]

SUPPLEMENTAL METHODS

X-ray irradiation

Pre-transplant conditioning was performed using an X-rad 320 irradiator (Precision X-ray, North Branford CT) powered by an Isovolt Titan E 320 (GE Sensing and Inspection Technologies, Lewiston PA). Doses were calibrated using a DMC 6000 Dose Measurement and Control Device (Precision X-ray). Settings were 320 kVolt and 12.5 mA. Filter 2 (0.8 mm Tin + 0.25 mm Copper + 1.5 mm Aluminum, half value layer ~3.7 mm Cu) was used for all irradiations. Mice were placed in a single layer in a plexiglass pie cage (Braintree Scientific, Braintree MA) followed by centering of the cage entirely within the X-ray field.

Immunofluorescence staining

Livers from mice receiving fl/fl versus AMPK dKO T cells were recovered on day 22 post-transplant, flash frozen in optimum cutting temperature (OCT) media, and sliced into 6 µm sections. Tissue was processed and stained using a modified protocol from the University of Pittsburgh Center for Biological Imaging. Sectioned tissues were fixed with a 1:1 methanol-acetone mixture for 15 minutes at -20 ºC. Following fixation, sections were washed 3 times with 0.5% BSA in PBS, blocked with 0.5% BSA/5% goat serum in PBS, washed 5 times, and incubated with primary antibodies in 0.5% BSA/5% goat serum PBS at 4 ºC overnight. Sections were washed 5 times the next morning, followed by incubation of secondary antibody for 45 minutes at room temperature. Sections were washed 5 times with 0.5% BSA in PBS, 5 times with PBS, then counterstained with DAPI (300 nm) for 3 minutes at room temperature. Three final washes in PBS were followed by mounting in Gelvatol (PVA, glycerol, and sodium azide) prior to coverslip application and sealing of the slide. Dual fluorescence staining was performed using a primary antibody against murine CD3e followed by Alexa-Fluor 488 conjugated anti-Armenian hamster IgG and a concurrent anti-CD68 primary antibody followed by secondary staining with Alexa-Fluor 647 conjugated goat anti-rabbit. Further antibody details in Supplementary Table 4.

Image acquisition and analysis:

Following staining, slides were imaged slides using a Zeiss LSM 710 confocal microscope. For each stained section, the ten most significant peri-portal infiltrates were identified and images acquired at 20x magnification. CellProfiler open-source software was then used to develop a cell counting pipeline based on an example pipeline from CellProfiler (see supplemental Table 5 for workflow). After optimization of the cell-counting pipeline via paired manual counting of representative images, the pipeline was used to count the number of CD3-positive cells in each high-powered field, as well as calculate the percentage of CD3-positive cells (from total DAPI cells) in each image acquired. For each sample, the three images with the highest cellular count were selected for statistical analysis. Samples from 8 recipients of wild type cells and 8 recipients of AMPK dKO cells were used. Thus, a total of 24 infiltrates for each experimental condition was included in the final statistical analysis. As noted elsewhere, Student paired t-tests detected statistical differences between infiltrates characteristics in recipients of wild type versus AMPK dKO T cells.

Treg suppression assay

$2x10^3$ $CD4^+CD25^{neg}$ T_{con} from a minimum of three independent biologic replicates were labeled with CellTrace Violet and plated with $2x10^3$ Mitomycin-C treated antigen presenting cells, 1 ug/ml anti-CD3 antibody, and varying ratios of fl/fl or AMPK dKO $CD4^+FoxP3^+$ T_{reg} from $FoxP3^{DTRGFP}$ mice. T_{con} "Division Index" was assessed at 72 hours, measuring the average number of divisions per cell and used to calculate percent suppression. To account for variance in T_{con} division between biological replicates, the division index of T_{con} at a particular T_{con}/T_{reg} ratio was divided by the baseline T_{con} division for that sample

(i.e. without T_{reg}). This fraction was then converted to a percentage and subtracted from 100 to achieve the final “% suppression”.

SUPPLEMENTAL FIGURES

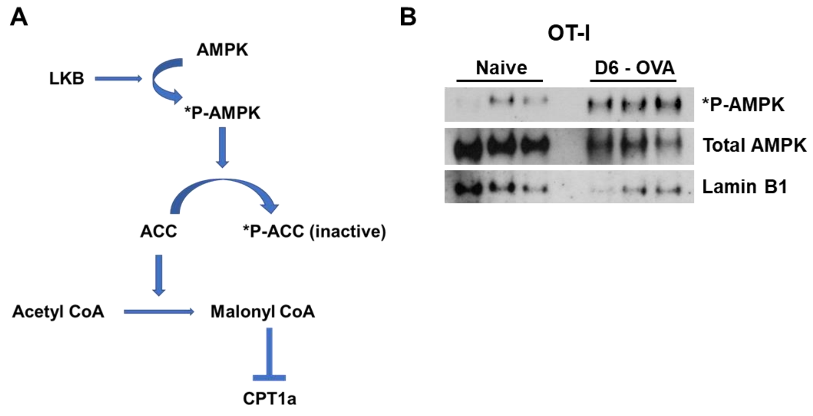

Supplemental Figure 1. Antigen-driven activation of AMPK. *A*, When intracellular AMP/ATP ratios rise, AMPKα becomes phosphorylated on residue Thr172 by liver kinase B1 (LKB), which increases AMPK's kinase activity. Activated AMPK then phosphorylates downstream targets including Acetyl-CoA Carboxylase (ACC) and Unc51-like kinase (ULK-1). In skeletal muscle, phosphorylation of ACC inhibits its carboxylase activity, decreasing malonyl-CoA production, liberating the allosteric inhibition of CPT1a, and thereby increasing mitochondrial lipid transport as a precursor to β-oxidation. *B*, 1×10^6 OT-I and 1×10^6 OT-II cells were transplanted into irradiated CAG-OVA mice. On day 6 post-BMT, OT-I cells were flow-sorted and immunoblotting performed as in Figure 1A, comparing phospho/total AMPK ratios in pre-transplant (naïve) OT-I T cells to those recovered from antigen-bearing recipients on day +6 (n=10 animals per condition pooled into 3 groups).

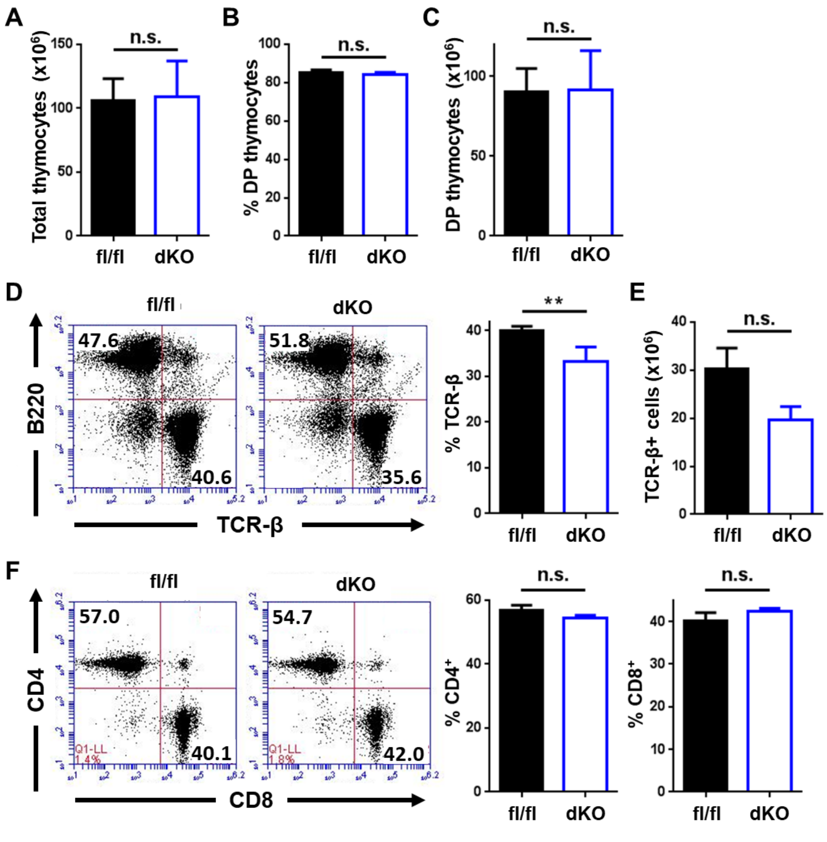

Supplemental Figure 2. Intact thymic and peripheral T cell development in the absence of AMPK. *A-C,* AMPKα1$^{fl/fl}$AMPKα2$^{fl/fl}$ mice were crossed to CD4-Cre animals and thymi recovered from naïve mice at eight weeks of age. Total thymocyte number (A), percentage of double positive (DP) thymocytes (B), and number of DP thymocytes (C) was evaluated by flow cytometry. *D-F,* Splenocytes were recovered from donors in A-C and the percentage of B220 versus TCR-β cells (D), total TCR-β^{+} cells (E), and CD4^{+} versus CD8^{+} percentages within the TCR-β^{+} gate (F) were quantitated. N= 4 animals/group and data represent two independent experiments. **p<0.01 by unpaired Student T test.

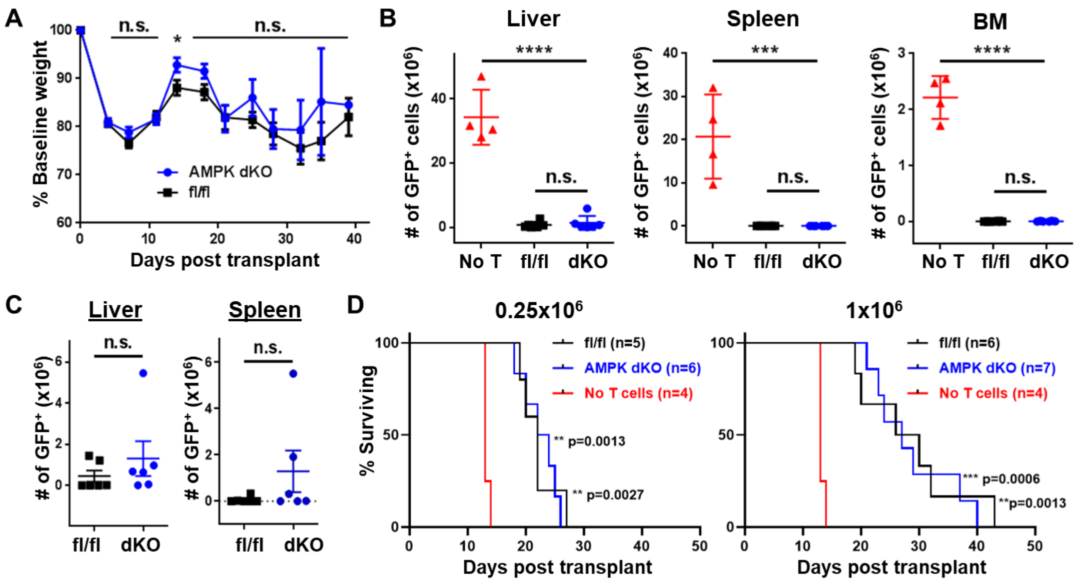

Supplemental Figure 3. AMPK dKO T cells mediate equivalent leukemia clearance. *A-C*, 1×10^6 fl/fl or AMPK dKO donor T cells were transplanted into irradiated B6D2F1 recipients with 5×10^6 TCD BM cells and 10^5 GFP$^+$ p815 leukemia cells (n= 15 mice/group). Recipients transplanted with only BM and p815 cells served as a positive control. Leukemia-bearing mice were weighed twice weekly and changes from baseline graphed as a function of time (A). On day 13 post-transplant, the total number of GFP$^+$ cells was quantitated in the liver, spleen, and BM of recipient mice (B). *C*, In a separate cohort, the total number of GFP$^+$ cells was quantitated in the liver and spleen of recipient animals on day 28, around the time of median overall survival)n= 6-7 mice/group). *D*, 1×10^6 or 0.25×10^6 fl/fl or AMPK dKO donor T cells were transplanted as above with 5×10^6 TCD BM cells and 10^5 GFP$^+$ p815 leukemia cells and recipient mice were monitored for survival out to 8 weeks post-transplant. P values in (D) represent comparisons to survival of no T cell control mice. *p<0.05, **p<0.01, ***p<0.001, ****p<0.0001. P value for survival curves was determined by log-rank (Mantel Cox) test. All other statistics were by Student T test.

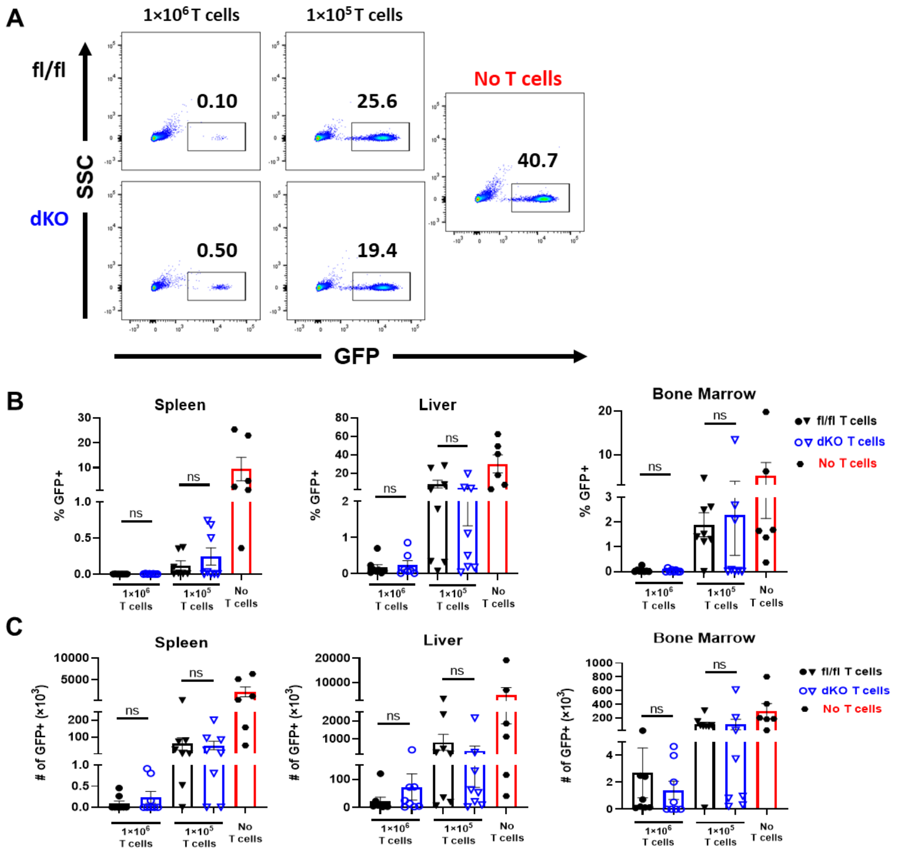

Supplemental Figure 4. Equivalent leukemia control with 10-fold fewer WT and AMPK dKO T cells. *A-C*, 1×10^6 or 1×10^5 donor T cells were transplanted as in Supplemental Figure 3D followed by quantitating the percentage and total number of GFP$^+$ cells in the spleen, liver, and BM of recipient mice on day 13 post-transplant (B, C). Representative flow plots for gating of GFP$^+$ cells are shown in (A). n= 6-8 mice/group.

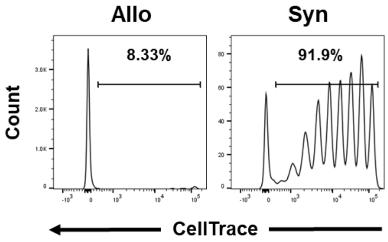

Supplemental Figure 5. Lower rates of cell division following syngeneic transplantation 2x10^6 CD45.1$^+$ B6 T cells were injected into lethally irradiated B6D2F1 (Allo) or B6 (Syn) recipients, recovered on day 7 post-transplant, and division profiles of CD45.1$^+$TCRβ$^+$ donor T cells assessed by flow cytometry.

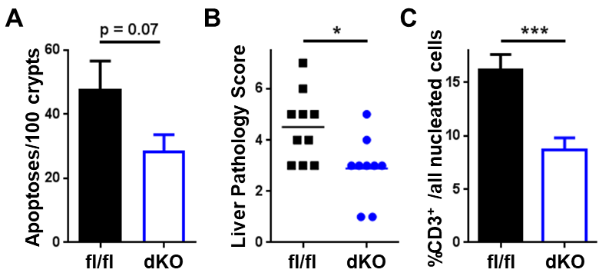

Supplemental Figure 6. Decreased target organ pathology in recipients of AMPK dKO cells. *A-B*, $2x10^6$ fl/fl or AMPK dKO T cells were transplanted into lethally irradiated B6D2F1 recipients and the number of apoptotic cells per 100 crypts was quantitated in hematoxylin and eosin (H&E) stained sections of small intestine removed on day 22 post-transplant (A). Livers were recovered from the same animals, stained with H&E, and scored for portal inflammation, bile duct injury, and central perivenulitis on a scale of 0 to 3 by a trained pathologist in a blinded fashion. These components were then totaled to generate an overall liver pathology score, with a max score of 9 (B). n= 10 mice/group in A/B. *C*, Computer-assisted confocal image analysis quantitated the percentage of $CD3^+$ cells (of all nucleated ($DAPI^+$) cells) in each high-powered field of freshly frozen livers collected from a separate cohort of B6D2F1 transplanted mice (n= 24 hpf/group). *p<0.05, ***p<0.001 by unpaired Student T test.

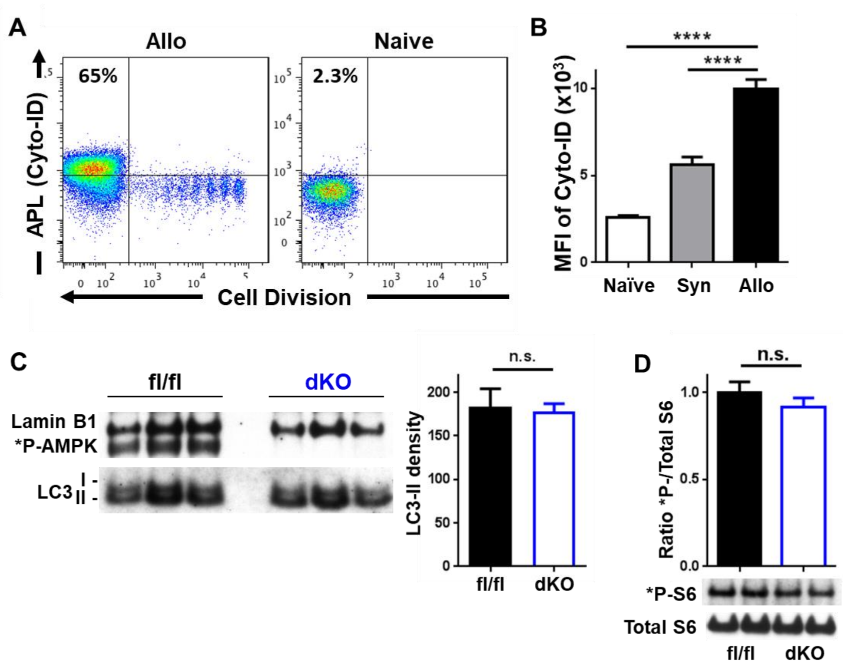

Supplemental Figure 7. Autophagy remains unchanged in allogeneic AMPK dKO T cells. *A-D*, 2x10^6 CD45.1$^+$ B6 T cells were left unmanipulated (naïve) or labeled with CellTrace violet and transplanted with 5×10^6 B6 BM cells into irradiated B6 (Syn) or B6D2F1 (Allo) recipients. On day 7 post-transplant, the percentage of donor T cells positive for Cyto-ID, a marker of autophagolysosome (APL) formation, was evaluated by flow cytometry (A) and the median fluorescence intensity (MFI) for Cyto-ID staining averaged in donor T cells from multiple recipients (B). fl/fl or AMPK dKO CD8 T cells were flow sorted from recipients on day 7 and cell lysates blotted for LC3-II formation (n= 10 mice pooled into 3 sets/group). *D*, fl/fl or AMPK dKO donor CD8$^+$ T cells were flow-sorted on day 7 and cell lysates immunoblotted for phospho- and total S6 protein levels (n=3-5 mice/group). ****p<0.0001 by Student T test.

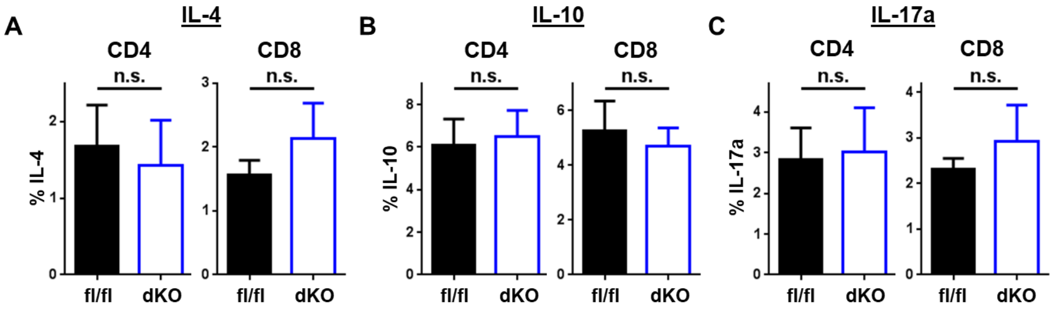

Supplemental Figure 8. No change in expression of Th2 or Th17 cytokines in AMPK dKO T cells. Transplantation was performed as in Figure 5C-D with fl/fl or AMPK dKO T cells recovered on day 7 post-transplant and stimulated with PMA and Ionomycin in the presence of Brefeldin A. After 4 hours, the percentage of IL-4^{+} (A), IL-10^{+} (B), and IL-17a^{+} (C) donor cells was determined by intracellular flow cytometry within the CD4^{+} and CD8^{+} subsets (n= 5-6 mice/group).

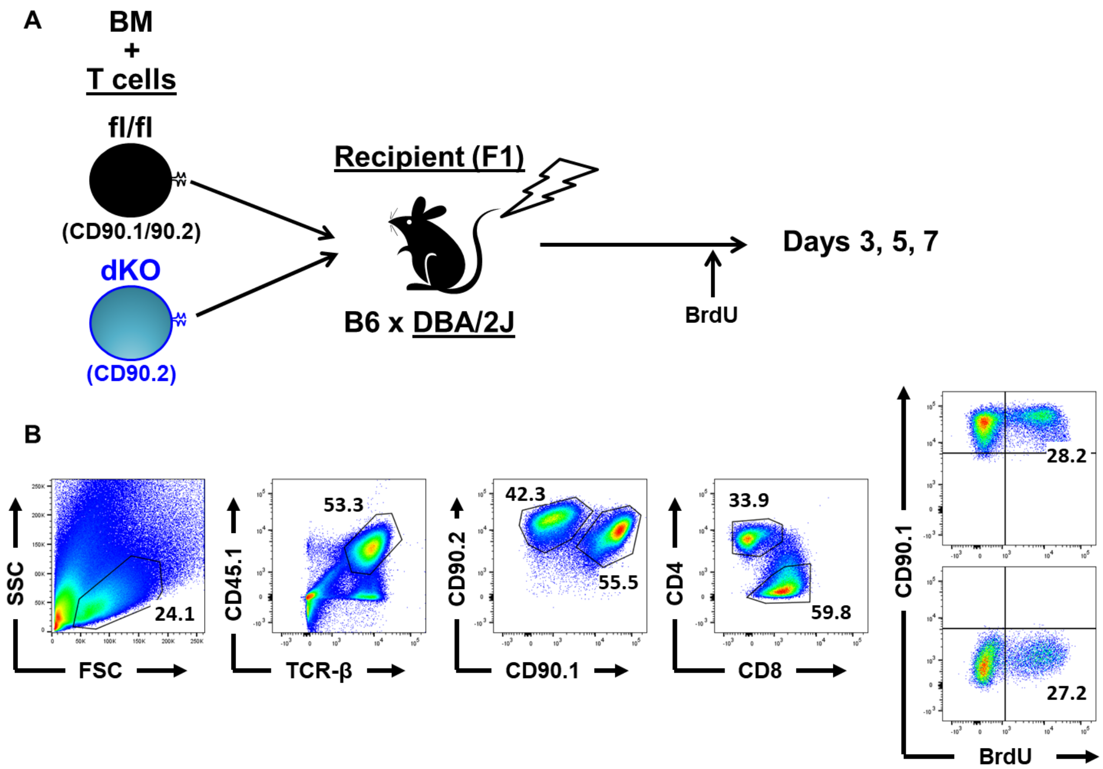

Supplemental Figure 9. Competitive transplantation of fl/fl versus AMPK dKO T cells. *A*, 1×10^6 fl/fl (CD45.1$^+$,CD90.1$^+$/CD90.2$^+$) and 1×10^6 AMPK dKO T cells (CD45.1$^+$,CD90.2$^+$) T cells were competitively transplanted into irradiated B6D2F1 recipients. BrdU was administered to recipient animals 30 minutes prior to euthanization on days 3, 5, and 7 post-transplant. *B*, gating strategy for cells transplanted in (A). From left to right, cells were gated as lymphocytes, then donor T cells (TCR-B$^+$CD45-1$^+$), and finally for CD90.2$^+$/CD90.1$^+$ (WT) versus CD90.2$^+$ (KO) status. To determine proliferation, fl/fl or AMPK dKO cells were further gated on CD4 versus CD8 positivity, followed by BrdU staining versus CD90.1 expression. Plots show a spleen sample harvested on day 5 post-transplant. Composite data from 8 mice/group is displayed in Figure 5E-H and Supplemental Figure 10.

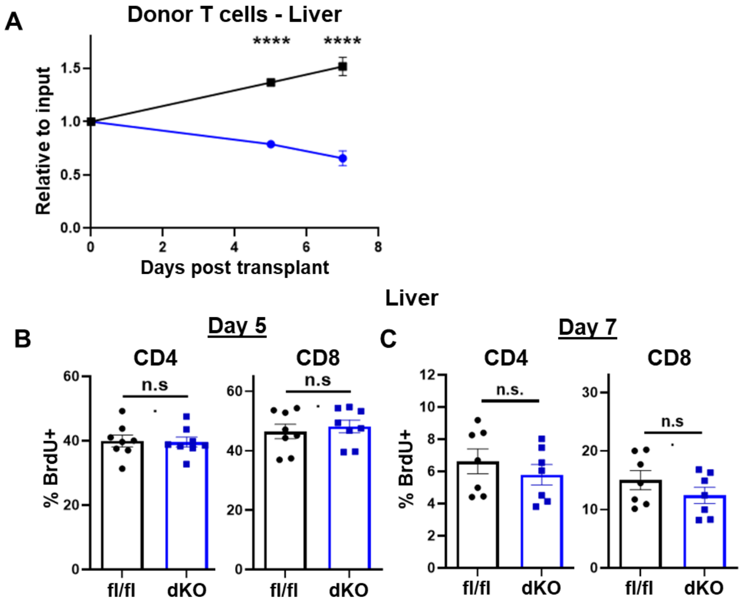

Supplemental Figure 10. Decreased numbers but equivalent division of liver-associated AMPK dKO T cells. *A-C*, 1×10[6] fl/fl donor T cells (CD90.1/CD90.2) were combined with 1×10[6] AMPK dKO T cells (CD90.2) and injected into irradiated B6D2F1 recipients. The percentage of fl/fl or AMPK dKO total T cells in recipient livers on days 5 and 7 post-transplant was compared input percentages (A). Recipients in (A) were injected with BrdU 30 minutes prior to euthanization and the percentage of BrdU$^+$ cells quantitated in liver-associated CD4 and CD8 donor T cells on day 5 (B) and day 7 (C) post-transplant. n= 7-8 mice/group, data represent ≥ 2 independent experiments. ****p<0.0001 by Student T test

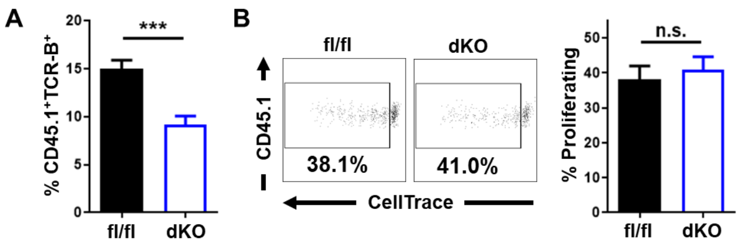

Supplemental Figure 11. Equivalent division of donor T cells on day 3 post-transplant. *A-B*, $2x10^6$ fl/fl or AMPK KO T cells were transplanted into individual B6D2F1 recipients and the percentage of CD45.1$^+$TCR-B$^+$ donor T cells quantitated on day 3 post-transplant (A). The percentage of T cell going into division by day 3 was then calculated (B). n= 6-8 mice/group, data represent ≥ 2 separate experiments. ***p<0.001 by unpaired Student T test

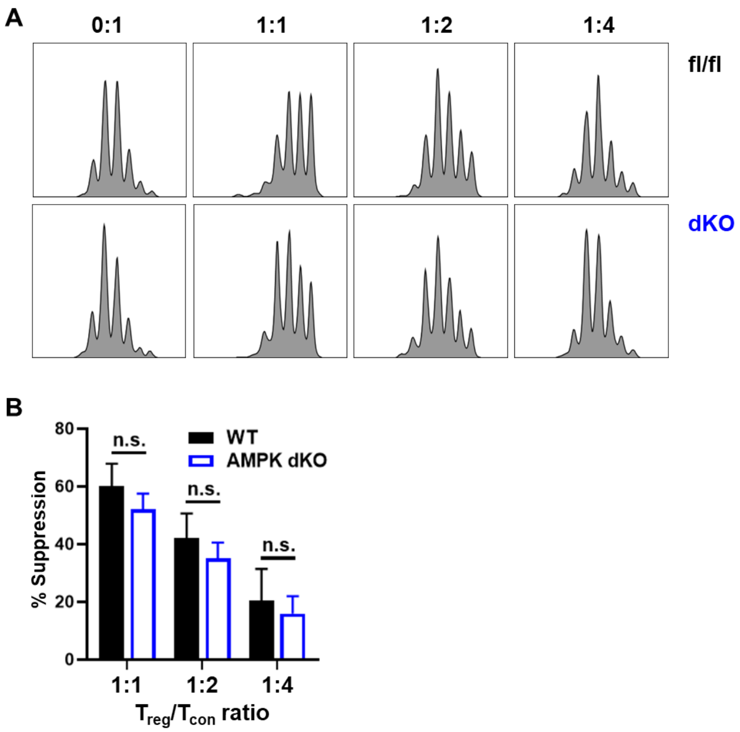

Supplemental Figure 12. Comparable suppressive function of AMPK dKO T_{reg}. *A-B,* CellTrace-labeled $CD4^+CD25^{neg}$ wildtype T_{con} were stimulated with anti-CD3 antibodies and co-cultured with Mitomycin-C treated APCs and varying ratios of fl/fl or AMPK dKO T_{reg}. $CD4^+$ T_{con} proliferation was assessed at 72 hours. Representative CellTrace profiles are shown in (A), with the percent suppression averaged from multiple biological replicates shown in (B). Data were obtained using T_{con} and T_{reg} from three independent donor animals per group and represent data from ≥3 individual experiments.

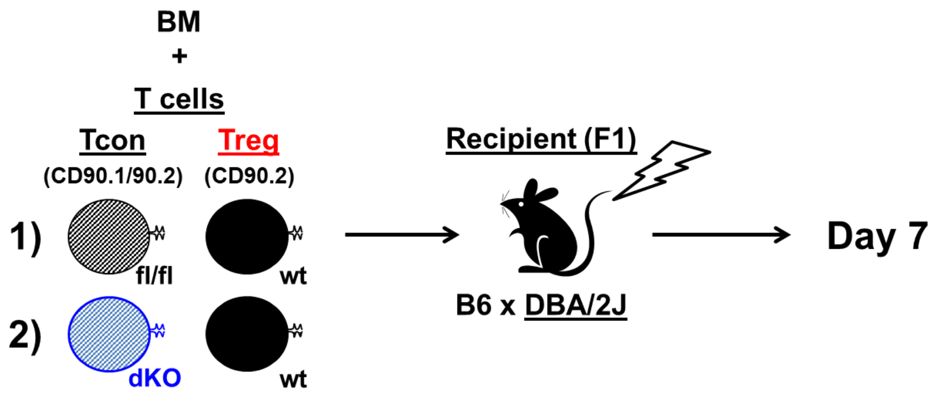

Supplemental Figure 13. Transplantation of wildtype T_{reg} with fl/fl or AMPK dKO T_{con}. Wildtype or AMPK dKO T cells (both $CD90.1^+/CD90.2^+$) were depleted of $CD25^+$ cells, added to wildtype $CD90.2^+$ T_{reg} (which totaled 15% of all $CD4^+$ cells), and transplanted into irradiated B6D2F1 recipients. On day 7, the percentage of $FoxP3^+$ cells, from within the total donor $CD4^+$ population, or specifically the original $CD4^+CD90.2^+$ T_{reg} population, was assessed by flow cytometry. Data are displayed in Figure 6C-D.

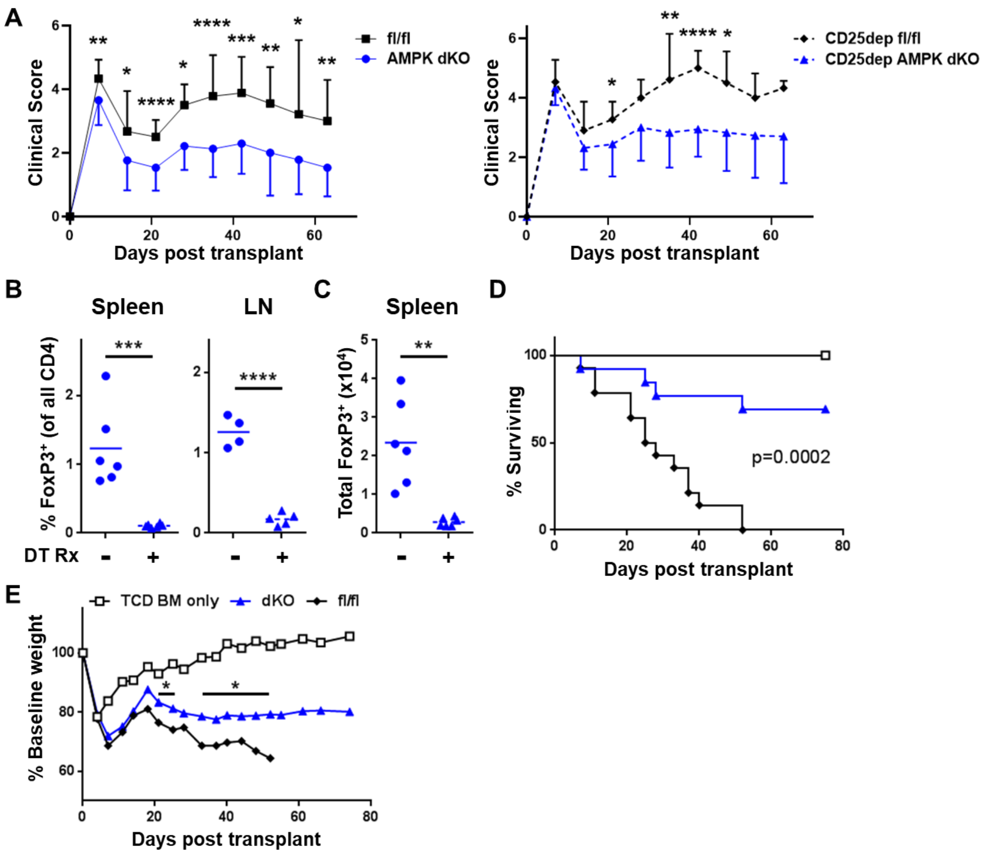

Supplemental Figure 14. Post-transplant depletion of T_{reg} does not eliminate AMPK KO-mediated benefits. *A*, Wildtype or AMPK dKO donor T cells, with or without pre-transplant depletion of $CD25^+$ cells, were injected into B6D2F1 recipients and clinical scores assessed weekly to 10 weeks post-transplant. Left panel compares clinical scores in recipients of fl/fl versus AMPK dKO donor cells without $CD25^+$ cell depletion, right panel depicts clinical scores with pre-transplant depletion of $CD25^+$ cells (n= 20 mice combined from two separate experiments). *B-C*, B6D2F1 recipients were transplanted with AMPK dKO_FoxP3^{DTR} donor T cells and administered 50 ng/kg diphtheria toxin (DT) on day 0 and +1 post-transplant. The percentage of FoxP3$^+$ cells in spleen/mesenteric LN (B) or the total number of FoxP3$^+$ cells in the spleen (C) was quantitated on day 7 (n= 4-6 mice/group, data represent 2 independent experiments). *D-E*, FoxP3^{DTR} fl/fl versus AMPK dKO donor T cells were transplanted into B6D2F1 recipients, treated on day 0 and +1 with DT, and followed for survival (D) and weight loss (E) to 10 weeks post-transplant (n=13-16 mice/group). Mice receiving TCD BM only served as a no-GVHD control (n=8).Asterisks in (E) refer to statistically significant differences between fl/fl and AMPK dKO groups. *p<0.05, **p<0.01, ***p<0.001, ****p<0.0001 by Student T test. P values for survival curves were determined by log-rank (Mantel Cox) test.

SUPPLEMENTAL TABLES

Supplementary Table 1 – Antibodies for Immunoblotting

Antigen	***Company***	***Catalog number***	***Clone***
ACC	*Cell Signaling*	*3676*	*C83B10*
Phospho-ACC (Ser172)	*Cell Signaling*	*11818*	*D7D11*
AMPKα	*Cell Signaling*	*2603*	*23A3*
AMPKα	*Cell Signaling*	*2793*	
Phospho-AMPKα (Th172)	*Cell Signaling*	*2535*	*40H9*
Lamin B1	*Cell Signaling*	*12586*	*D4Q43*
LC3 A/B	*Cell Signaling*	*12741*	*D3U4C*
LKB	*Cell Signaling*	*3047*	*D60C5*
Phospho-ULK1 (Ser555)	*Cell Signaling*	*5869*	*D1H4*
S6 Ribosomal protein	*Cell Signaling*	*2217*	*5G10*
Phospho-S6	*Cell Signaling*	*2215*	*Polyclonal*

Abbreviations: ACC – Acetyl-CoA Carboxylase, AMPK – AMP-activated protein kinase, LC3 - Light Chain 3, LKB – Liver kinase B1, ULK-1 – Unc51-like kinase 1.

Supplementary Table 2 – Antibodies for flow cytometry

Flow cytometry antibodies				
Antigen	***Conjugate***	***Company***	***Clone***	***Catalog Number***
B220	*FITC*	*BD Biosciences*	*RA3-6B2*	*553088*
CD4	*FITC, PE*	*Invitrogen (Thermo Fisher)*	*RM4.4*	*11-0043-85*
CD4	*PE*	*BD Biosciences*	*RM4-5*	*553049*
CD8	*eFluor780*	*Invitrogen*	*53-6.7*	*47-0081-82*
CD45-1	*APC, APC-eFluor 780*	*Invitrogen*	*A20*	*17-0453-82* *47-0453-82*
CD45-1	*BV711*	*BioLegend*	*A20*	*110739*
CD90.1	*PerCP-Cy5.5*	*Invitrogen*	*HIS51*	*45-0900-82*
CD90.2	*PE-Cy7*	*Invitrogen*	*53-2.1*	*25-0902-82*
FoxP3	*FITC, PE, APC, PE-Cy7*	*Invitrogen*	*FKJ-16s*	*11-5773-82* *12-5773-80* *17-5773-82* *25-5773-82*
IFNγ	*PE, APC*	*Invitrogen*	*XMG1.2*	*12-7311-82* *17-7311-81*
IL-4	*PE*	*Invitrogen*	*11B11*	*12-7041-81*
IL-10	*PE*	*Invitrogen*	*JES5-16E3*	*12-7107-81*
IL-17a	*PE*	*Invitrogen*	*eBio17B7*	*12-7177-81*
TCR-β	*PerCPCy5.5*	*Invitrogen*	*H57-597*	*45-5961-82*
TNFα	*APC*	*Invitrogen*	*MP6-XT22*	*17-7321-81*

Supplementary Table 3 – Reagents for flow cytometry

Additional flow cytometry reagents			
Reagent	***Ex/Em***	***Company***	***Catalog Number***
Annexin-Alexa Fluor488	*488/499*	*Invitrogen*	*A13201*
CellTrace Violet	*405/450*	*Invitrogen*	*C34557*
Cyto-ID	*359, 460/527*	*Enzo Life Sciences*	*51031*
Streptavidin eFluor 450	*405/450*	*Invitrogen*	*45-5961-82*

Supplementary Table 4 – Reagents for immunofluorescence

Antigen	***Conjugate***	***Company***	***Clone***	***Catalog Number***
Primary antibodies				
CD3e	*Purified*	*Invitrogen*	*145-2C11*	*14-0031-82*
CD68	*Purified*	*Abcam*	*Polyclonal*	*125212*
Secondary antibodies				
Armenian hamster IgG	*Alexa Fluor 488*	*Jackson ImmunoResearch Laboratories*	*Polyclonal*	*127-545-160*
Rabbit IgG	*Alexa Fluor 647*	*Jackson ImmunoResearch Laboratories*	*Polyclonal*	*111-605-144*
Other reagents				
DAPI (4′,6-diamidino-2-phenylindole)	*None*	*Invitrogen*	*N/A*	*D1306*

Supplemental Table 5 – CellProfiler workflow

Module	Function	Settings/Parameters
ColorToGray	Split RGB image into respective colors and each color channel to convert to grayscale image	Output images specified OrigiGreen, OrigiBlue, and OrigiRed for later analysis
IdentifyPrimaryObject	Identify and select nuclei from OrigiBlue image	Diameter between 10 and 40 pixels, threshold correction factor of 1, two class Otsu threshold, intensity used to distinguish clumped objects
OverlayOutlines	Draw outline around nuclei identified in previous step and save image to verify accuracy	Red outlines on blue objects
IdentifyPrimaryObject	Identify and select CD3 positive cells from OrigiGreen image	Diameter between 10 and 50 pixels, threshold correction factor of 1with lower limit of 0.1 and upper limit of 1, two class Otsu threshold, intensity used to distinguish clumped objects
OverlayOutlines	Draw outline around CD3-positive object identified in previous step and save image to verify accuracy	Red outlines on green objects
RelateObjects	Pair nuclei with neighboring CD3 positive objects	Nuclei as parent object, CD3 as child object
ClassifyObjects	Classify paired and unpaired nuclei by CD3 status	Nuclei as input object to be classified, CD3 objects as measurement by which to classify, output as "some" CD3 object vs. "no" CD3 object
FilterObjects	Filter classified nuclei by intensity of associated CD3 measurement for final statistical analysis	Nuclei as input, CD3 count as filter, minimum measurement defined as 1 with no maximum, output as number of CD3-positive nuclei
CalculateMath	Calculate the percentage of nuclei associated with a CD3-positive object to obtain percentage of CD3-positive cells per image	CD3 positive nuclei count as numerator, nuclei count as denominator
ExportToSpreadsheet	Save calculated data to Excel spreadsheet	
